# Ottogi Inhibits Wnt/β-catenin Signaling by Regulating Cell Membrane Trafficking of Frizzled8

**DOI:** 10.1038/s41598-017-13429-6

**Published:** 2017-10-16

**Authors:** Hyun-Taek Kim, Mi-Sun Lee, Yun-Mi Jeong, Hyunju Ro, Dong-Il Kim, Yong-Hwan Shin, Ji-Eun Kim, Kyu-Seok Hwang, Jung-Hwa Choi, Minjin Bahn, Jeong-Ju Lee, Sang H. Lee, Young-Ki Bae, Jin-Soo Lee, Joong-Kook Choi, Nam-Soon Kim, Chang-Yeol Yeo, Cheol-Hee Kim

**Affiliations:** 1 Department of Biology, Chungnam National University, Daejeon, 34134 South Korea; 2 Department of Life Science and Research Center for Cellular Homeostasis, Ewha Womans University, Seoul, 120-750 South Korea; 3 Genome Research Center, Korea Research Institute of Bioscience and Biotechnology, Daejeon, 305-806 South Korea; 4 Department of Pharmacology & Toxicology, Medical College of Wisconsin, Milwaukee, WI 53226 USA; 5 National Cancer Center, Goyang, 410-769 South Korea; 6 Department of Biochemistry, College of Medicine, Chungbuk National University, Cheongju, 361–763 South Korea

## Abstract

Wnt signaling controls critical developmental processes including tissue/body patterning. Here we report the identification of a novel regulator of Wnt signaling, *OTTOGI (OTG)*, isolated from a large-scale expression screening of human cDNAs in zebrafish embryos. Overexpression of *OTG* in zebrafish embryos caused dorso-anteriorized phenotype, inhibited the expression of Wnt target genes, and prevented nuclear accumulation of β-catenin. Conversely, knockdown of zebrafish *otg* using specific antisense morpholino promoted nuclear accumulation of β-catenin and caused ventralization. However, OTG failed to rescue *headless*-like phenotype induced by inhibition of GSK-3β activity, suggesting that OTG acts upstream of GSK-3β. OTG bound specifically to Frizzled8 (Fz8) receptor and caused retention of Fz8 in the endoplasmic reticulum possibly by preventing N-linked glycosylation of Fz8. Taken together, our data indicate that OTG functions as a novel negative regulator of Wnt signaling during development by the modulation of cell surface expression of Fz receptor.

## Introduction

Wnt signaling pathway regulates multiple developmental processes, adult tissue homeostasis and stem cell renewal^1–3^. In addition, it has been implicated in various pathological conditions in humans such as neurodegenerative diseases and cancer. During early development, Wnt signals regulate cell fate decision, cell movement/migration, and tissue patterning. In the canonical Wnt pathway, Wnt protein binds to its cognate receptor complex consisting of a serpentine receptor of the Lrp5/6 and Frizzled (Fz) family. Ligand-receptor binding activates Dishevelled (Dvl) which subsequently disrupts the so-called “destruction complex” containing APC, GSK-3β and Axin. In the absence of Wnt signaling, GSK-3β constitutively phosphorylates β-catenin, leading to proteosomal degradation of β-catenin^4,5^. Upon Wnt stimulation, unphosphorylated β-catenin can localize to the nucleus where it binds to transcription effector Tcf/Lef and activates the transcription of target genes through the recruitment of various cofactors.

Several negative regulators of Wnt signaling pathway have been reported. Secreted proteins including secreted Fz-related proteins (sFRPs), Wnt inhibitory protein (WIF), and Dickkopf (DKK) function as Wnt antagonists for both canonical and non-canonical Wnt signaling pathways by sequestering Wnt ligands or competitively binding to Lrp5/6^2^. In the cytosol, Axin1/2, APC and GSK-3β can inhibit Wnt signaling by promoting β-catenin degradation^6^. In *Xenopus*, the ER protein Shisa inhibits maturation/glycosylation of Fz receptors and prevents their cell surface trafficking^7^. The Fz family proteins encode seven transmembrane G-protein coupled receptors (GPCRs). Most GPCRs are N-linked glycoproteins containing an Asn-X-Ser/Thr motif within extracellular domains^8^. N-glycosylation of GPCRs reduces denaturation/proteolysis, enhances solubility, and facilitates correct orientation of proteins relative to the plasma membrane^9^. Fz receptors also contain several potential N-glycosylation sites whose glycosylation acts positively in Wnt signaling transduction^10,11^.

We carried out a large-scale expression screening of human genes in zebrafish embryos. More than 10,000 full-length human cDNA clones were obtained from the Korean UniGene Information System and non-characterized genes were arbitrarily selected for screening. Proteins were overexpressed individually in zebrafish embryos and examined for their ability to induce morphological defects. We tested over 2,700 cDNA clones and identified several genes of interest. Affected zebrafish embryos displayed various morphological defects; from among them, we report on a novel function of *OTTOGI* (*OTG*) in anterior-posterior body patterning.

*OTG*/*Prr7* was also identified from a postsynaptic density (PSD) fraction of rat hippocampal neurons and was reported to bind PDZ domains of PSD-95^12^. Recently, PRR7 has been characterized as a transmembrane adaptor protein that is up-regulated in activated human peripheral blood lymphocytes and involved in T-cell receptor signaling. PRR7 overexpression in T cell lines leads to apoptosis by triggering c-Jun up-regulation^13^. However, the role of *OTG*/*Prr7* during development has yet to be reported. Our results indicate that OTG regulates anterior-posterior patterning of zebrafish embryos, acting as a negative regulator of Wnt signaling.

## Results

### Large-scale expression screening of full-length human cDNAs in zebrafish

**Figure 1:**
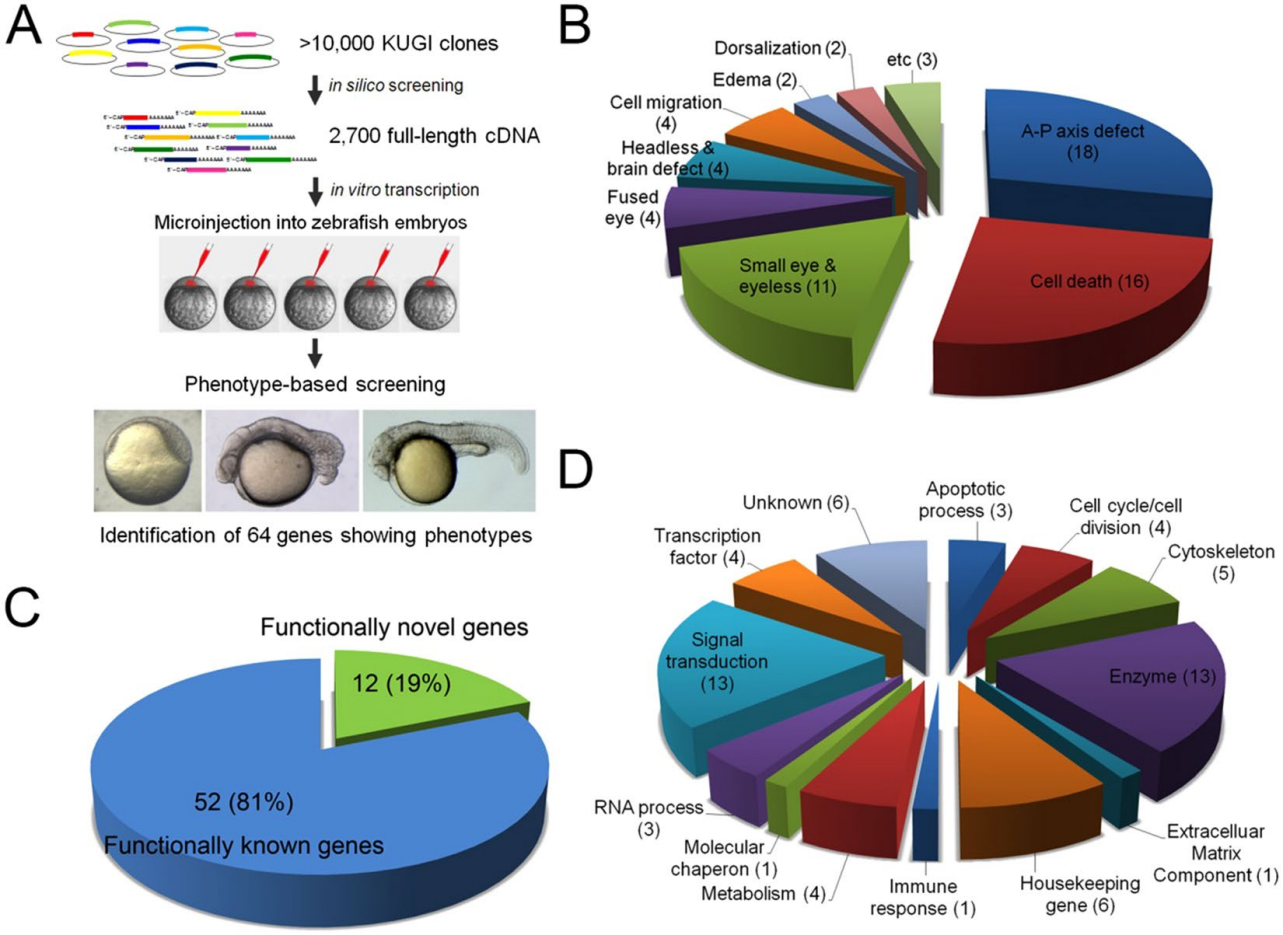
Schematic diagram of results from a large-scale expression screening of human full-length cDNAs. (**A**) Synthetic mRNA for individual human full-length cDNA clone was injected to one to two-cell stage zebrafish embryos. Injected embryos were examined for morphological defects from early embryogenesis (6 hpf) to organogenesis (24–48 hpf). Total of sixty-four genes caused morphological defects. (**B**) Types of defects identified from the expression screening. Numbers in parentheses indicate the number of genes that caused particular defect. (**C,D**) Using bioinformatics tools including PROSITE, SMART and Motif Scan programs, identified genes are classified by biological processes they regulate.

To identify human genes with novel function, a large-scale expression screen was performed in zebrafish for the human unigene collection. Over 10,000 full-length human cDNAs were subjected to database search for function and 2,700 genes of unknown function were tested by injecting synthetic mRNAs into zebrafish embryos. The effects on embryonic development were assessed by monitoring morphological defects from shield (6 hpf) to long-pec (2 dpf) stages. Of the genes tested, sixty-four genes caused various defects during development (Fig. 1).

**Figure 2:**
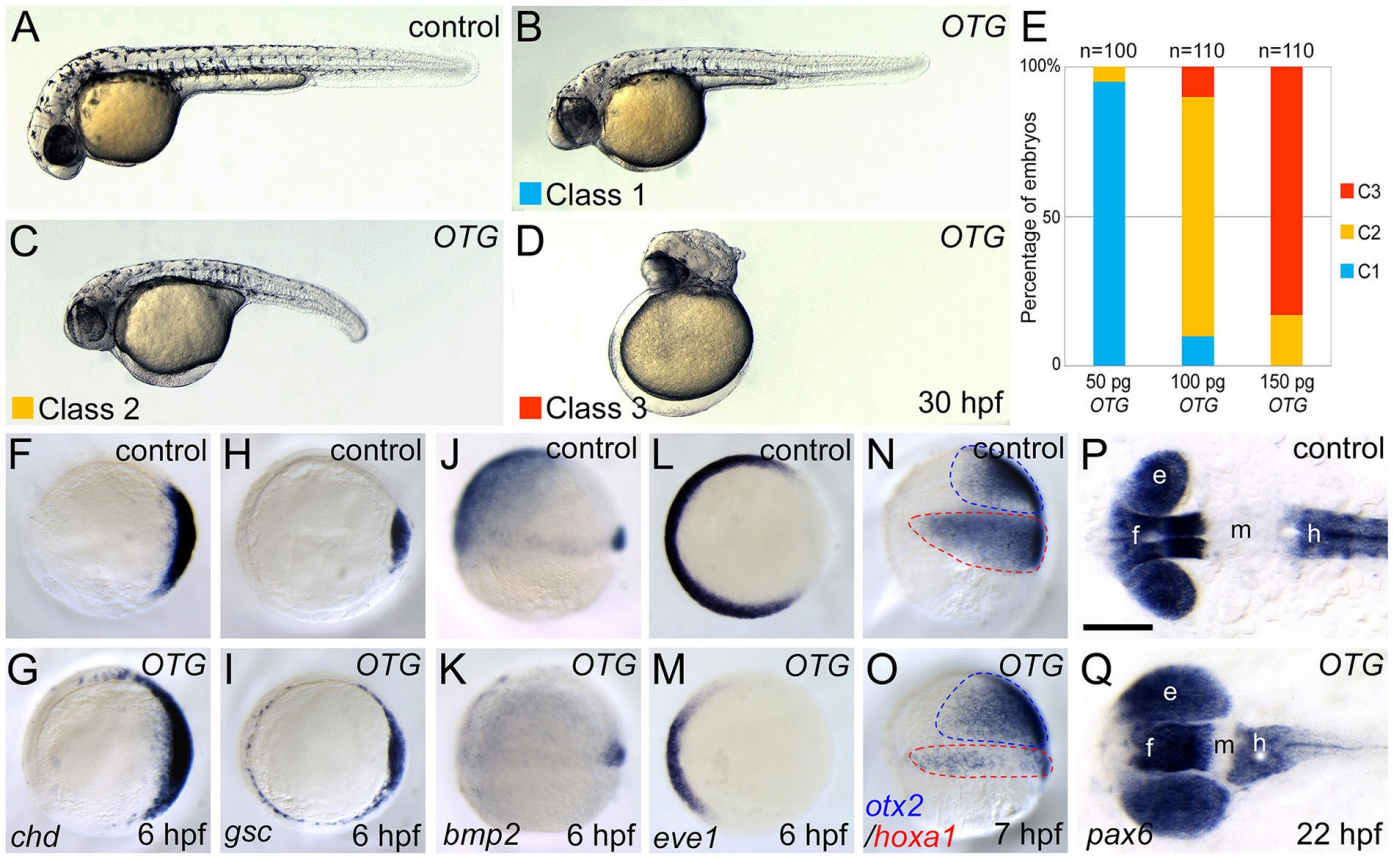
OTG overexpression causes dorsoanteriorized phenotypes in zebrafish embryos. (**A**) Un-injected control embryo. (**B–D**) Phenotypes of *OTG* mRNA-injected embryos were classified into three classes (see text). (**E**) Frequencies of phenotypes caused by injection of indicated amounts of *OTG* mRNA. (**F–O**) Views from animal pole, dorsal to the right (**F**–**I**,**L**,**M**). Lateral view (**J**,**K**,**N**,**O**). Expression patterns of shield markers *chd* (F, 20/20, 100%; G, 13/15, 87%) and *gsc* (H, 15/15, 100%; I, 14/15, 93%), a ventral marker *bmp2* (J, 12/12, 100%; K, 11/12, 92%), a ventral mesoderm marker *eve1* (L, 10/10, 100%; M, 11/12, 92%), and anterior neural marker *otx2* and a posterior neural marker *hoxa1* (N, 12/12, 100%; O, 15/16, 94%) in un-injected control embryos (**F**,**H**,**J**,**L**,**N**) or *OTG* mRNA-injected embryos (**G**,**I**,**K**,**M**,**O**) at indicated developmental stages. (**P,Q**) Dorsal views, anterior to the left. Expression pattern of *pax6* in un-injected control (P, 20/20, 100%) or *OTG* mRNA-injected (Q, 14/16, 87%) embryo at 20-somite stage (22 hpf). Abbreviations. e, eye; f, forebrain; h, hindbrain; m, midbrain.

We focused our analysis on the functionality of clone 462 which encodes the 274 amino acids-long human PRR7 (Supplementary Fig. S1). Clone 462 caused an enlarged head with truncated tail possibly due to defects in dorsoventral (D-V) and anterioposterior (A-P) patterning during early development (Fig. 2A–E). In order to avoid confusion with the plant *Prr* (*pseudo response regulator*) gene, we renamed this gene “*OTTOGI*” (meaning “roly-poly toy” in Korean) because of the resemblance of the overexpression phenotype to a roly-poly toy.

### OTG Promotes Head Formation during Embryo Development

Overexpression of OTG in developing embryos caused head enlargement and tail shortening. To analyze the overexpression phenotype in detail, human *OTG* mRNA was injected into zebrafish embryos at various doses. The resulting defects were categorized into three classes based on their severity: Class I (Fig. 2B), embryos with enlarged head but no distinguishable axial defect; Class II (Fig. 2C), embryos with enlarged head and shortened body axis; Class III (Fig. 2D), embryos with enlarged head without any discernible axial structure. Severity of defects was found to increase in a dose-dependent manner (Fig. 2E). We isolated OTTOGI genes from human, mouse, and zebrafish. We also tested the activity of these genes using overexpression analysis and found no significant differences in their activity as a negative regulator of Wnt signaling (Supplementary Fig. S2).

Because OTG overexpression resulted in embryos resembling those with mutations in genes critical for dorsoventral (D-V) and anterioposterior (A-P) patterning, we examined the expression of D-V and A-P patterning markers in *OTG* mRNA-injected embryos. At the onset of gastrulation, expression domains of dorsal organizer markers such as *chd* (87%, n = 15; Fig. 2F,G) and *gsc* (93%, n = 15; Fig. 2H,I) were expanded ventro-laterally at the expense of ventral markers such as *bmp2* (92%, n = 12; Fig. 2J,K), *bmp4* (93%, n = 15; Supplementary Fig. S3A,B), and *eve1* (92%, n = 12; Fig. 2L,M). In addition, expression domains of anterior neuroectoderm markers such as *otx2* (94%, n = 16; Fig. 2N,O) and *tlc/sfrp5* (86%, n = 15; Supplementary Fig. S3C,D) were also expanded; whereas, those of posterior neuroectoderm marker *hoxa1* (94%, n = 16; Fig. 2N,O) were reduced. The expression domain of pan-neural marker *sox2* was also expanded ventro-laterally (92%, n = 13; Supplementary Fig. S3E,F). However, the expression patterns of endoderm markers *wnt11* and *mixer* were not affected at gastrula stage (Supplementary Fig. S3G–J). To examine A-P patterning of the CNS, expression of forebrain and hindbrain marker *pax6* was analyzed. OTG overexpression shifted the forebrain expression domain of *pax6* more posteriorly at the expense of midbrain (87%, n = 16; Fig. 2P,Q). These results indicate that OTG overexpression caused dorsalization and anteriorization during early embryonic development.

### OTG negatively regulates Wnt/β-catenin pathway

**Figure 3:**
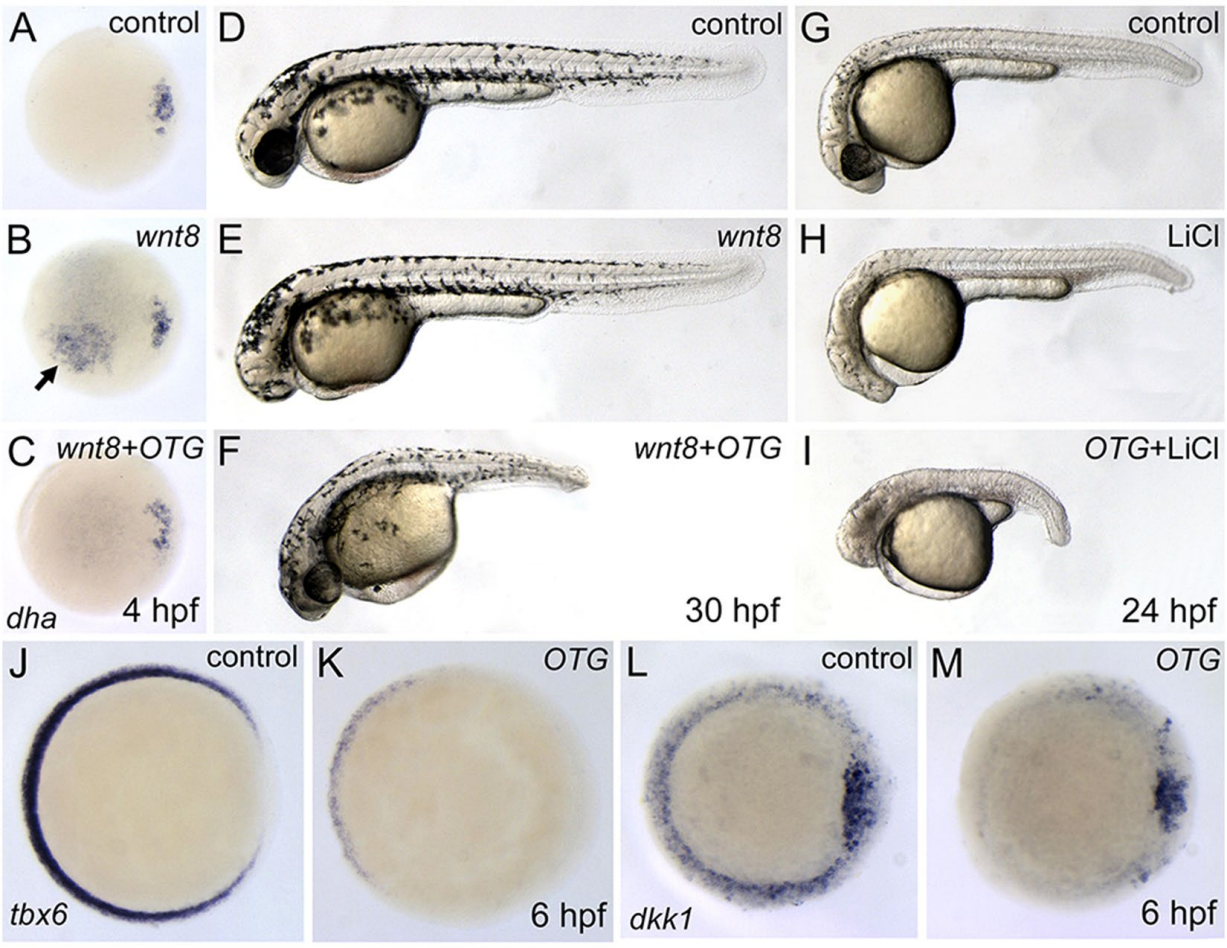
OTG inhibits Wnt/β-catenin signaling pathway during early development. (**A–C**) Views from animal pole, dorsal to the right. Expression pattern of *dha* in control (A, 10/10, 100%), *wnt8* mRNA-injected (B, 14/15, 93%), or *wnt8* and *OTG* mRNA-injected embryo (C, 12/13, 92%) at sphere stage (4 hpf). Arrow indicates Wnt8-induced ectopic expression of *dha*. (**D**–**F**) Morphology of control (D, 10/10, 100%), *wnt8* mRNA-injected (E, 18/20, 90%), or *wnt8* and *OTG* mRNA-injected embryo (F, 17/20, 85%) at 30 hpf. (**G**–**I**) Morphology of control (G, 12/12, 100%), LiCl treated (H, 13/15, 86%), or LiCl treated and *OTG* mRNA-injected embryo (I, 14/15, 93%) at 24 hpf. (**J–M**) Views from animal pole, dorsal to the right. Expression pattern of *tbx6* (J, 10/10, 100%; K, 18/20, 90%) or *dkk1* (L, 10/10, 100%; M, 17/20, 85%) in control (**J**,**L**) or *OTG* mRNA-injected (**K**,**M**) embryo at 6 hpf.

During gastrulation, interplay between Wnt inhibitors and Wnts establishes a gradient of Wnt/β-catenin activity which regulates A-P patterning of gastrula^14,15^. Because Wnt signaling inhibitors, including *dkk1* and *sfrps*, are known to inhibit secondary axis formation induced by ectopic Wnt8^16,17^, we examined whether OTG can inhibit Wnt8. Targeted injection of *wnt8* mRNA into single cells at 4–8 cell stages led to ectopic expression of *dharma* (93% n = 15; Fig. 3A,B) and *chordin* (80%, n = 10; Supplementary Fig. S4A,B) which are target genes of canonical Wnt signaling pathway^18,19^. Overexpression of OTG abolished Wnt8-induced ectopic expression of *dharma* (92%, n = 13; Fig. 3C) and *chordin* (83%, n = 12; Supplementary Fig. S4C).

To position OTG in the Wnt/β-catenin pathway, we performed epistatic analysis. Ectopic expression of Wnt8 has been shown to lead to a *headless*-like phenotype such as eye defects and a small head^20^. At 30 hpf, *wnt8* mRNA-injected embryos developed with compromised forebrain structures (90%, n = 20; Fig. 3E). However, co-injection of *OTG* mRNA suppressed Wnt8-induced *headless*-like phenotype (85%, n = 20; Fig. 3F), suggesting that OTG negatively regulates the Wnt/β-catenin pathway and may act downstream of Wnt8.

Next, we tested whether OTG counteracts β-catenin. Overexpression of β-catenin leads to secondary body axis formation. Interestingly, however, overexpression of OTG failed to suppress β-catenin-induced secondary body axis (93% n = 14; Supplementary Fig. S4D–F). VP16-tcf3, a dominant negative form of Tcf3, leads to *headless*-like phenotype by transcriptional activation of Wnt target genes^21^. OTG did not suppress the VP16-tcf3-induced *headless*-like phenotype (89%, n = 18; Supplementary Fig. S4G–I). In addition, OTG failed to rescue *headless*-like phenotype induced by GSK3β inhibitor LiCl (93%, n = 15; Fig. 3G–I) and SB415286 (78%, n = 18; Supplementary Fig. S4J–L). These results suggest that OTG acts upstream of β-catenin and GSK3β.

We also examined whether OTG inhibits expression of endogenous Wnt target genes such as *tbx6* and *dkk1* ^22,23^. At shield stage, the expression domains of *tbx6* (90%, n = 20; Fig. 3J,K) and *dkk1* (85%, n = 20; Fig. 3L,M) were dramatically reduced in OTG overexpressed embryos.

### Isolation and expression profile of Zebrafish *otg*

Zebrafish *otg* encodes a 245 amino acid-long polypeptide, having high similarity to human, mouse, and *Xenopus* proteins (Supplementary Fig. S1). From RT-PCR analysis, *otg* is expressed maternally and zygotically (Supplementary Fig. S5A). Whole-mount *in situ* hybridization indicated that *otg* transcripts are ubiquitously distributed before the onset of somitogenesis (Supplementary Fig. S5B–E). As somitogenesis progresses, *otg* transcripts are restricted to the anterior region (Supplementary Fig. S5F,G). At 24 and 36 hpf, *otg* transcripts were detected in the brain and the anterior region of the pronephric duct (Supplementary Fig. S5H, I). At 36 hpf, *otg* is expressed bilaterally in the proximal convoluted tubule (PCT) of the pronephros (Supplementary Fig. S5J). At 72 hpf, *otg* transcripts were detected in the brain, pharyngeal arch, and PCT of the pronephros (Supplementary Fig. S5K). In the adult brain, *otg* transcripts were detected in distinct regions (Supplementary Fig. S5L).

### Loss of *otg* results in ventralized phenotypes

In order to assess the physiological roles of zebrafish *otg*, we first verified the specificity of *otg* MO. First, *otg* MO was co-injected with *otg-GFP* fusion mRNA. Embryos injected with *otg-GFP* mRNA alone showed GFP fluorescence at the 4-somite stage whereas *otg* MO suppressed GFP fluorescence (93%, n = 15; Supplementary Fig. S6D,E). However, *otg* MO failed to suppress GFP expression from *otgΔN-GFP* mRNA lacking MO-targeting sequences (100%, n = 13; Supplementary Fig. S6F). Second, we performed a rescue experiment by co-injecting *otg* MO with *otg* mRNA. *otg* MO alone caused expanded intermediate cell mass (ICM) with a dwindled head, but *OTG* or *otg* mRNA co-injection alleviated the morphological defects (87%, n = 16; Supplementary Fig. S6G–I). The fact that human *OTG* rescued zebrafish morphants suggests conservation of Otg function across vertebrate species.

**Figure 4:**
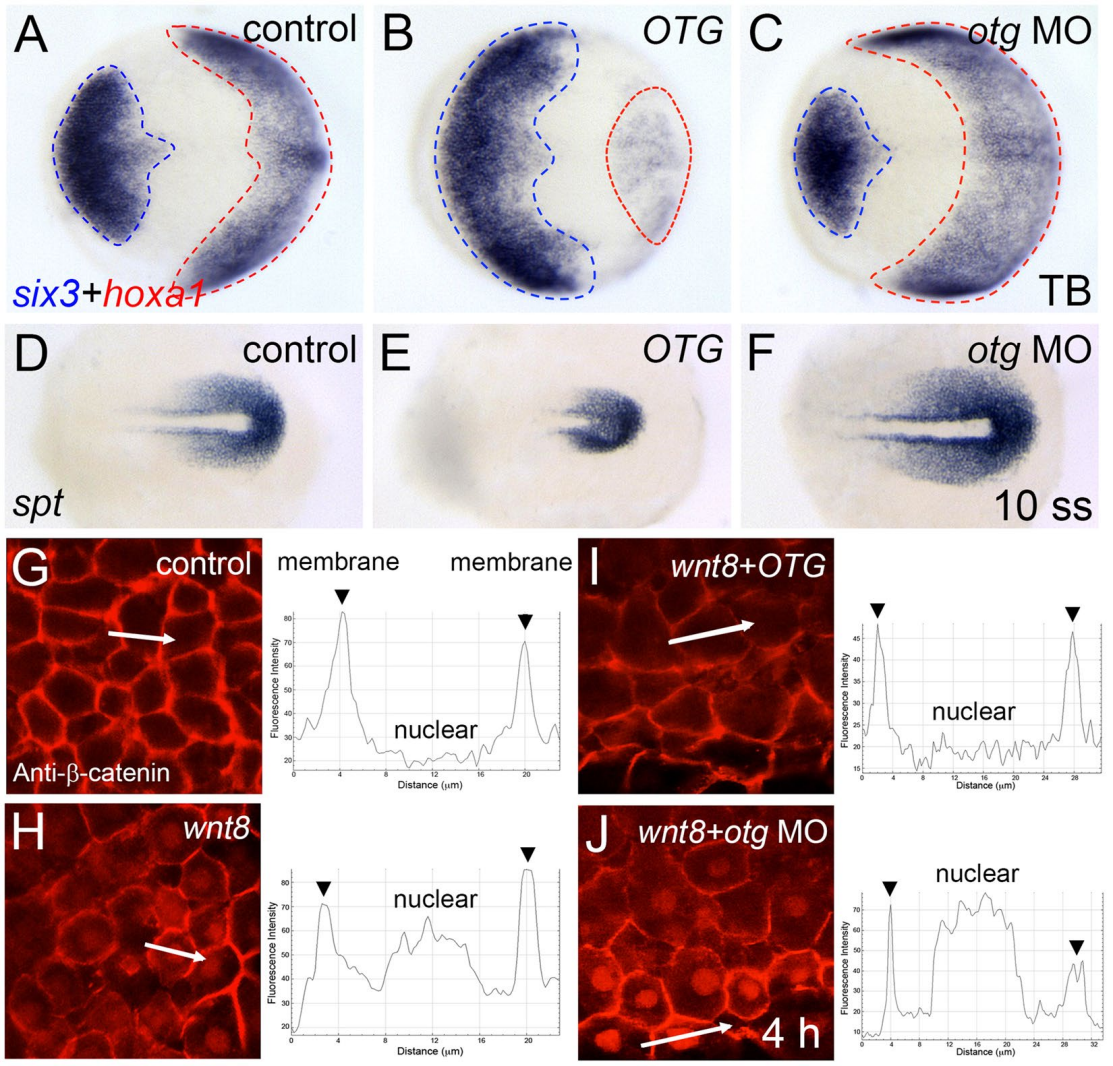
Loss of *otg* function affects AP-DV patterning and nuclear β-catenin localization. (**A–C**) Doral views, anterior to the left. Expression patterns of an anterior neural marker *six3* and a posterior neural marker *hoxa1* in control (A, 12/12, 100%), *OTG* mRNA-injected (B, 17/20, 85%) or *otg* MO-injected (C, 16/18, 89%) embryo at tail bud stage (10 hpf). (**D–F**) Doral views, posterior to the right. Expression pattern of a posterior mesoderm marker *spt* in control (D, 10/10, 100%), *OTG* mRNA-injected (E, 18/20, 90%) and *otg* MO-injected (F, 20/23, 87%) embryo at 10 somite stage. (**G–J**) Views of animal pole cells at blastula stage. Immunostaining for β-catenin and fluorescence intensity profile along the white lines in control (**G**), *wnt8* mRNA-injected (**H**), *wnt8* and *OTG* mRNA-injected (**I**), or *wnt8* mRNA and *otg* MO-injected (**J**) embryo.

*otg* morphants displayed a reduced head and enlarged ICM (92%, n = 25; Supplementary Fig. S6A–C). This phenotype is similar to that of *chordino* mutants^24^. To characterize the ventralized phenotype in detail, we examined several relevant markers. At neural plate stage (10 hpf), OTG overexpression led to the expansion of *six3*-expressing anterior neuroectoderm with reduction of *hoxa1*-expressing posterior neuroectoderm (85%, n = 20; Fig. 4A,B). In contrast, knockdown of *otg* caused an expansion of the posterior neuroectoderm at the expense of anterior neuroectoderm (89%, n = 18; Fig. 4C). Expression domains of *zic/opl* (90%, n = 20) and *rx1* (91%, n = 22), presumptive eye markers, were significantly reduced in *otg* morphants (Supplementary Fig. S6J–O). However, the posterior expression domain of *spt*, a pre-somitic mesoderm marker, was expanded in *otg* morphants but reduced in OTG-overexpressing embryos (90%, n = 20; Fig. 4D–F). Collectively these results suggest that *otg* is critical for the specification of normal D-V axis and possibly for fine-tuning of the canonical Wnt signaling pathway.

Nuclear accumulation of β-catenin in the canonical Wnt pathway is important for the expression of Wnt target genes^6^. Nuclear accumulation of β-catenin restricted to the dorsal blastula is a hallmark of the formation of dorsally located Organizer. Ectopic Wnt8 expression induces ectopic nuclear localization of β-catenin^25^. We examined β-catenin nuclear localization after overexpression or knock-down of *otg*. In animal pole cells of control embryos, β-catenin was localized at the plasma membrane (Fig. 4G). Ectopic Wnt8 induced nuclear accumulation of β-catenin (Fig. 4H). Wnt8-induced nuclear localization of β-catenin was abolished by OTG overexpression, but nuclear localization could still be observed in *otg* morphants (Fig. 4I,J).

Next, we examined the effects of OTG in a Wnt reporter [TOPdGFP] transgenic zebrafish^26^. In control embryos at shield stage, reporter activity was observed in the ventrolateral margin and shield hypoblast. However, expression was dramatically compromised in human *OTG* or zebrafish *otg* mRNA-injected embryos (Supplementary Fig. S7A–C). Conversely, *otg* MO increased reporter activity in the ventrolateral marginal zone (Supplementary Fig. S7D). Overexpression of Wnt8 enhanced [TOPdGFP] reporter expression (80%, n = 15; Supplementary Fig. S7E) whereas elevated reporter expression was significantly reduced in *OTG*/*otg* mRNA co-injected embryos (95%, n = 13/86%, n = 14; Supplementary Fig. S7F,G). In contrast, *otg* MO further enhanced Wnt8-induced reporter expression (83%, n = 12; Supplementary Fig. S7H). Taken together, these results indicate that Otg negatively regulates the Wnt/β-catenin pathway during early embryogenesis.

### OTG prevents cell surface expression of Fz8 through ER retention

**Figure 5:**
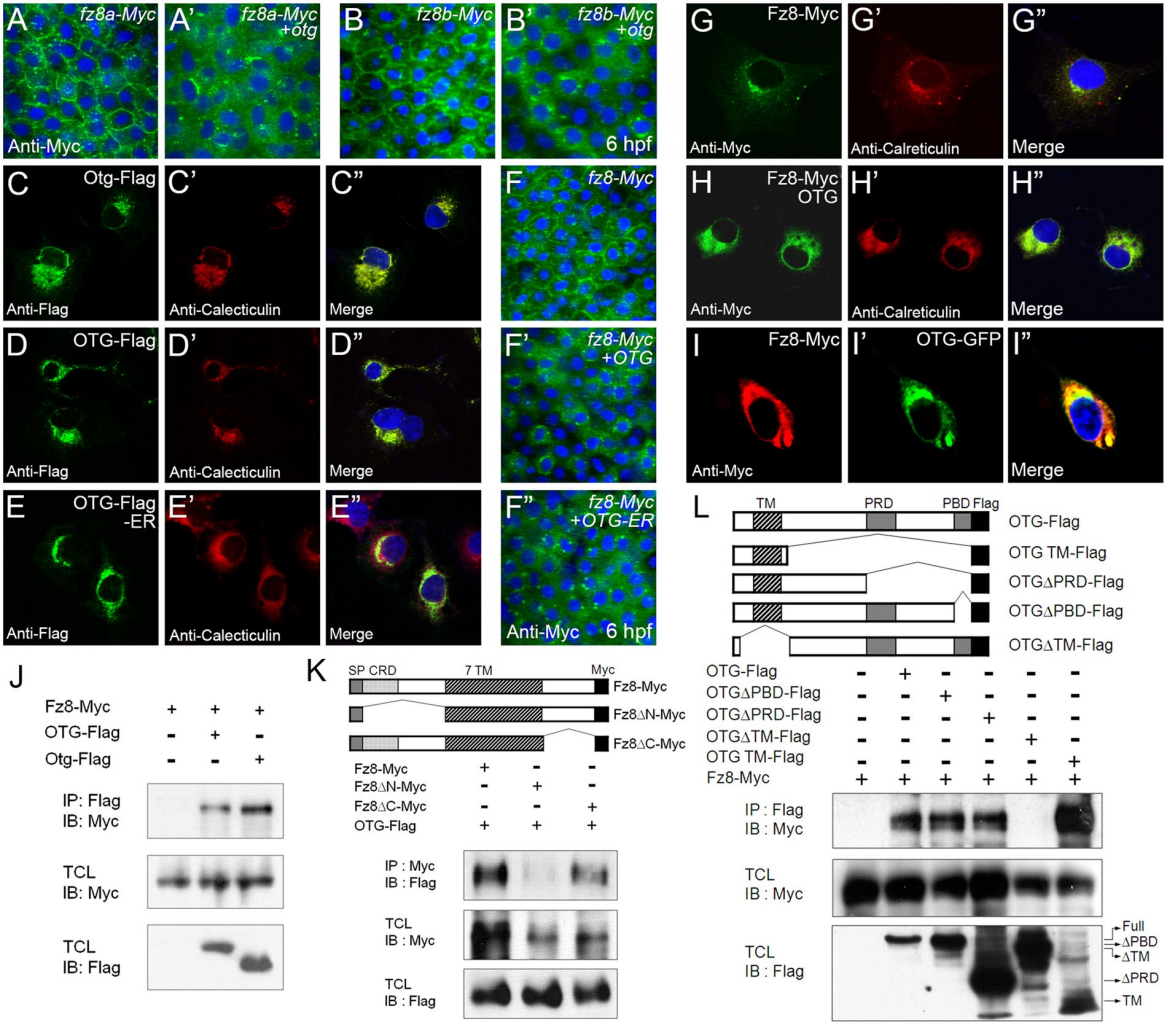
OTG prevents membrane targeting of Fz8 receptor and restricts Fz8 to the ER. (**A,B**) Immunostaining of Myc (green) in embryos (6 hpf) injected with indicated combinations of *otg*, *fz8a-myc* and *fz8b-myc* mRNAs. For panels A–I, nuclei are counter-stained with Hoechst 33342 (blue). (**C–E**) COS-7 cells were transfected for C-terminal Flag-tagged zebrafish Otg (C), human OTG (D), or human OTG with C-terminal fusion of an ER retention sequence KDEL (OTG-Flag-ER, E). Cells are immunostained for Flag (green) and an ER marker calreticulin (red). (**F**) Immunostaining of Myc (green) in embryos (6 hpf) injected with indicated combinations of *OTG*, *OTG-ER* and *fz8-myc* mRNAs. (**G,H**) COS-7 cells were transfected for Fz8-Myc alone (H) or with OTG (I). Cells are immunostained for Myc (green) and calreticulin (red). (**I**) NIH3T3 cells were transfected for Fz8-Myc and OTG-GFP. Cells are immunostained for Myc (red) and GFP (green). (**J–L**) Schematic representations of various deletion constructs are shown at the top of each panel. HEK293 cells were transfected for indicated combinations of OTG (OTG-flag), Otg (Otg-Flag) and Fz8-Myc. Interaction between Fz8 and OTG/Otg is examined by immunoprecipitation for OTG/Otg (IP: Flag) or Fz8 (IP: Myc) followed by immunoblotting for Fz8 (IB; Myc) or OTG (IB: Flag). Levels of proteins in total cell lysates (TCL) are also compared. Full-length blots are presented in Supplementary Fig. S19.

OTG acts downstream of the Wnt ligand, meaning that OTG may act downstream or in parallel with Fz receptor. We examined whether OTG affected subcellular localization of the Fz8 receptor. In zebrafish, whole-genome duplication results in two *fz8* gene paralogues; *fz8a* and *fz8b* ^27^. The majority of Fz8a-Myc and Fz8b-Myc localized at cell surface but Otg dramatically decreased cell surface expression (Fig. 5A,B). This suggests that Otg may diminish Wnt signaling by interfering with Fz8 membrane targeting. We also examined whether Otg affected subcellular localization of other Wnt signaling components, such as Lrp and Dvl. Lrp6-HA was localized in the plasma membrane and cytoplasmic vesicles; Otg did not affect subcellular localization of Lrp6-HA (Supplementary Fig. S8A,B). Cytoplasmic localization of Myc-Dvl1 was also not affected by Otg (Supplementary Fig. S8C,D). To test whether OTG acts specifically on Fz8, we examined another Fz receptors, Fz3 and Fz10. Cell surface localization of Fz3 and Fz10-Myc was not affected by OTG (Supplementary Fig. S8E–H) indicating specific alteration of subcellular Fz8 localization and not all Fz receptors. We also examined the effects of OTG on receptors for other signaling pathways critical for early embryonic patterning and found that OTG did not affect localization of BMP receptor Bmpr1 or FGF receptor Fgfr (Supplementary Fig. S8I–L).

In order to understand how OTG affects Fz8 membrane targeting, we analyzed the subcellular localization of OTG. Generally, OTG protein is localized in the ER in zebrafish embryos and cultured cells (Fig. 5C,D, Supplementary Fig. S9A,B”). Examination of the membrane topology of OTG suggests that OTG is a type II transmembrane protein in which the C-terminal domain faces the extracellular side (Supplementary Fig. S9C,D **)**. Many ER-resident proteins share a carboxyl-terminal Lys-Asp-Glu-Leu (KDEL) sequence and C-terminal fusion of the KDEL tetrapeptide to a secreted protein is sufficient for ER retention^28^. OTG-Flag-KDEL (called OTG-ER hereafter) was exclusively localized in the ER (Fig. 5E). Overexpression of *OTG-ER* caused enlarged head and truncated tail similar to wild-type *OTG* (92%, n = 24; Supplementary Fig. S10A–C). At gastrulation, the expression domain of *chd* was dramatically expanded in either *OTG* or *OTG-ER* mRNA-injected embryos (85%, n = 13; Supplementary Fig. S10D–F). Moreover, OTG-ER prevented membrane targeting and induced ER retention of Fz8-Myc like wild type OTG (Fig. 5F). These results suggest that OTG function for Fz8 localization is confined to the ER.

Deletion analyses demonstrated that the N-terminus of OTG, including the transmembrane domain, is critical for ER retention (Supplementary Fig. S11; see Fig. 5L for schematic representation of OTG deletion mutants). C-terminal deletions (OTGΔPRD-GFP and OTGΔPBD-GFP) did not alter ER localization of OTG (Supplementary Fig. S11D,E”), and the majority of OTG TM-GFP, containing only extracellular and transmembrane domains, was still localized in the ER. However, a deletion mutant lacking N-terminal region (OTGΔTM-GFP) was found to be accumulated in the nucleus (Supplementary Fig. S11B,C”). These results suggest that N-terminal extracellular and transmembrane domains of OTG are necessary for its retention in the ER.

### OTG interacts with Fz8 receptor

To determine whether OTG interacts with Fz8, we first examined the co-localization of OTG with Fz8 in cultured cells. When Fz8-Myc was expressed alone, only a portion of Fz8-Myc overlapped with ER markers, such as calreticulin (Fig. 5G) and ER-ECFP (Supplementary Fig. S12A-A”). In contrast, when co-expressed with OTG, the majority of Fz8-Myc was localized in the ER (Fig. 5H and Supplementary Fig. S12B-B”). Moreover, OTG-GFP co-localized with Fz8-Myc in the cytoplasm particularly at perinuclear regions (Fig. 5I). Taken together, these results indicate that OTG prevents membrane targeting of Fz8 receptors by inducing ER retention, in both cultured cells as well as in zebrafish embryos.

Next, we mapped the domain(s) important for OTG-induced ER retention of Fz8 using various Fz8 deletion mutants. Fz8ΔC-Myc lacking cytoplasmic domain, was distributed largely in the cytoplasm and, for the most part, did not co-localize with calreticulin (Supplementary Fig. S13A-A”). However, when co-expressed with OTG, Fz8ΔC-Myc was accumulated in the ER (Supplementary Fig. S13B-B”). Fz8ΔN-Myc lacking extracellular domain was also mainly localized in the cytoplasm and did not co-localize with calreticulin (Supplementary Fig. S13C–C”). Unlike Fz8-Myc and Fz8ΔC-Myc, however, Fz8ΔN-Myc was not accumulated in the ER when co-expressed with OTG (Supplementary Fig. S13D-D”), indicating that the N-terminal region of Fz8 is required for OTG-induced ER-retention of Fz8 in cultured cells.

Next, we investigated by co-immunoprecipitation whether OTG interacts with Fz8 in cultured cells. OTG-Flag or Otg-Flag co-immunoprecipitated with Fz8-Myc to demonstrate OTG and Fz8 interaction (Fig. 5J, Supplementary Fig. S14A). Deletion of Fz8 C-terminal region did not prevent interaction with OTG; whereas, deletion of the N-terminal extracellular domain of Fz8 reduced it significantly (Fig. 5K). The Fz8 binding domain of OTG was mapped using various OTG deletion mutants (Fig. 5L). All OTG deletion mutants, except OTGΔTM-Flag interacted with Fz8-Myc. Moreover, N-terminal extracellular and transmembrane domains of OTG (OTG TM-Flag) were sufficient for Fz8 interaction. These results suggest that the extracellular domain of Fz8 and N-terminal region (including TM domain) of OTG mediate the interaction between OTG and Fz8.

We also examined the effects of OTG on membrane trafficking of Fz8 deletion mutants in zebrafish embryos. At onset of gastrulation, co-expression of OTG inhibited cell surface expression of both Fz8ΔC-Myc and Fz8ΔCRD-Myc (Supplementary Fig. S14A,B′). The CRD domain of Fz8 is located immediately after the signal peptide (see Fig. 5K); however, OTG failed to alter subcellular localization of Fz8ΔN-Myc (Supplementary Fig. S14C-C′) suggesting that Fz8 binds OTG through its N-terminal region but that CRD domain is not critical for this interaction.

We then analyzed whether OTG deletion mutants are also capable of inducing enlarged head as is wild type OTG. OTGΔPBD-Flag induced an enlarged head and truncated tail (94%, n = 16; Supplementary Fig. S15F). In contrast, OTG TM-Flag (71%, n = 14) and OTGΔPRD-Flag (75%, n = 16) resulted in some defects while OTGΔTM-Flag (73%, n = 15) did not cause any discernible phenotype (Supplementary Fig. S15C–E). Consistently, OTGΔPBD-Flag inhibited membrane expression of Fz8-Myc whereas OTG TM-Flag, OTGΔTM-Flag and OTGΔPRD-Flag did not (Supplementary Fig. S15G–L). These results indicate that PDZ-binding motif (PBD) of OTG may be dispensable for its Wnt antagonizing activity.

### OTG is involved in glycosylation of Fz8

**Figure 6:**
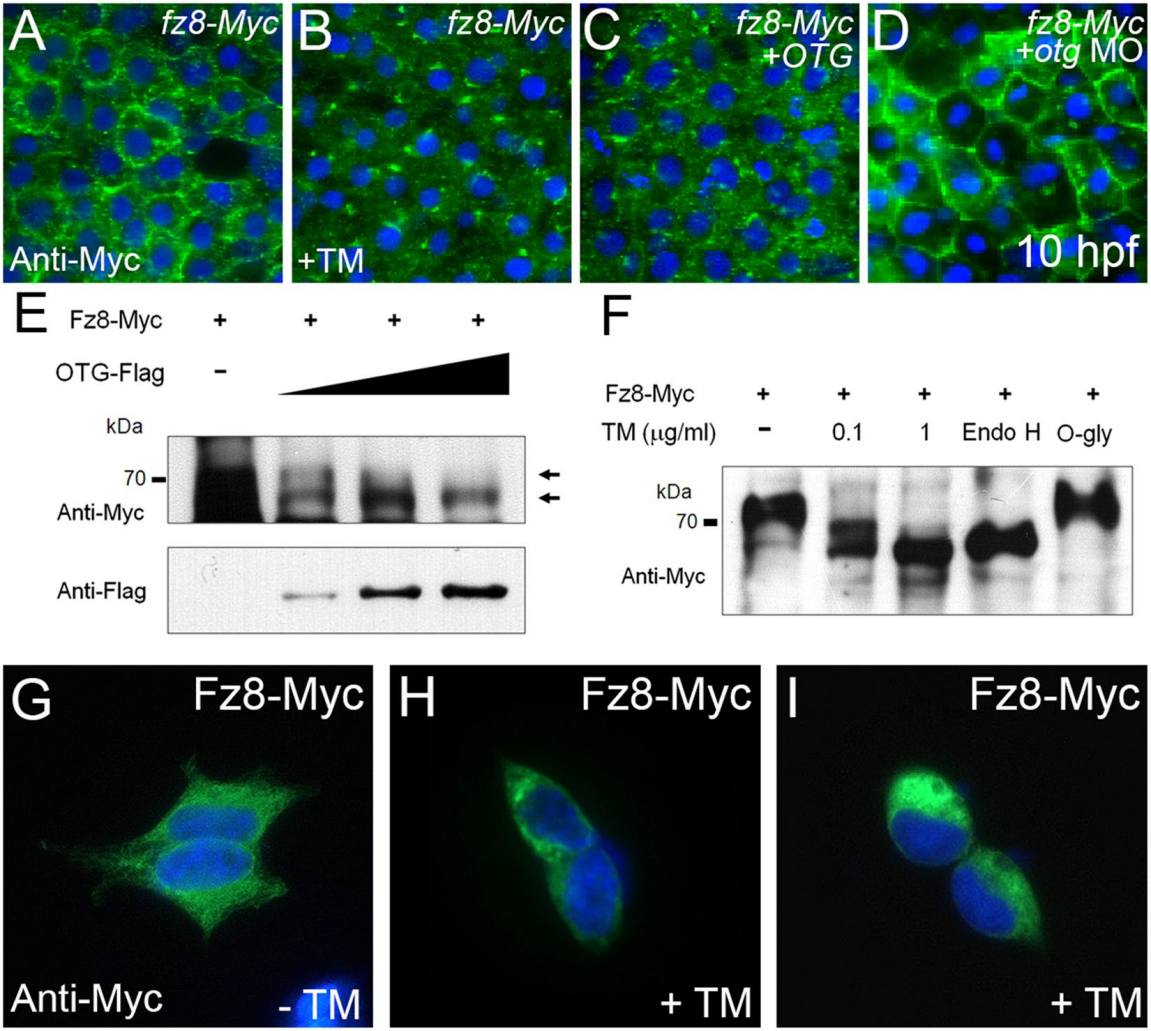
OTG regulates glycosylation of Fz8 receptor. (**A–D**) Immunostaining of Myc (green) in embryos (10 hpf) injected with *fz8-myc* mRNA alone (**A**), with *OTG* mRNA (**C**), with *otg* MO (**D**) or treated with an N-glycosylation inhibitor tunicamycin (**B**). For panels A–D and G–I, nuclei are counter-stained with Hoechst 33342 (blue). (**E**) HEK293 cells were transfected for Fz8-Myc and increasing amounts of OTG-Flag. Overexpressed Fz8-Myc (anti-Myc) and OTG-Flag (anti-Flag) proteins are analyzed by immunoblotting. (**F**) HEK293 cells were transfected for Fz8-Myc and then treated with tunicamycin, or their lysates were incubated with endoglycosidase H (Endo H) or O-glycosidase (O-Gly). Overexpressed Fz8-Myc protein (anti-Myc) is analyzed by immunoblotting. Full-length blots are presented in Supplementary Fig. S19. (**G**–**I**) HEK-293 cells were transfected with Fz8-Myc and then treated with vehicle DMSO alone (**G**) or with tunicamycin [0.1 μg/ml (H) and 1 μg/ml (**I**)]. Cells are immunostained for Myc (green).

Frizzled receptors contain several potential glycosylation sites which are critical for the onset of Wnt signaling by facilitating cell surface localization of Fz8^10,11^. Glycosylation of proteins begins in the ER; from there, glycosylated proteins are further modified in the Golgi complex before leaving for their final destinations. Since OTG interacts and co-localizes with Fz8 in the ER, we speculated that OTG may influence glycosylation and maturation of Fz8. The localization of Fz8 was examined in zebrafish embryos after treatment of tunicamycin (TM), an N-linked glycosylation inhibitor^29^. Similar to the effect of OTG on Fz8, TM treatment inhibited membrane expression of Fz8-Myc (Fig. 6A–C). In contrast, membrane expression of Fz8-Myc was enhanced in *otg* MO-injected embryos (Fig. 6D). These results suggest the possibility that inhibition of Fz8 glycosylation by Otg prevents membrane targeting of Fz8.

We also tested whether OTG affected glycosylation of Fz8. When overexpressed in cultured cells, electrophoretic mobility of Fz8-Myc protein was increased by OTG in a dose-dependent manner (Fig. 6E). Moreover, a similar mobility shift of Fz8-Myc was observed after treatment with TM or endoglycosidase H, an enzyme which cleaves oligosaccharides from N-linked glycoproteins (Fig. 6F). However, O-glycosidase, which catalyzes the removal of O-linked disaccharides, did not affect electrophoretic mobility of Fz8-Myc. Similar to TM-treated embryos, TM-treated cultured cells exhibited a distribution of Fz8-Myc which was restricted to the perinuclear region (Fig. 6G–I). These results suggest the possibility that OTG inhibits cell surface localization of Fz8 by interfering with N-linked glycosylation of Fz8.

### Glycosylation of Fz8 is required for Wnt/β-catenin signaling

**Figure 7:**
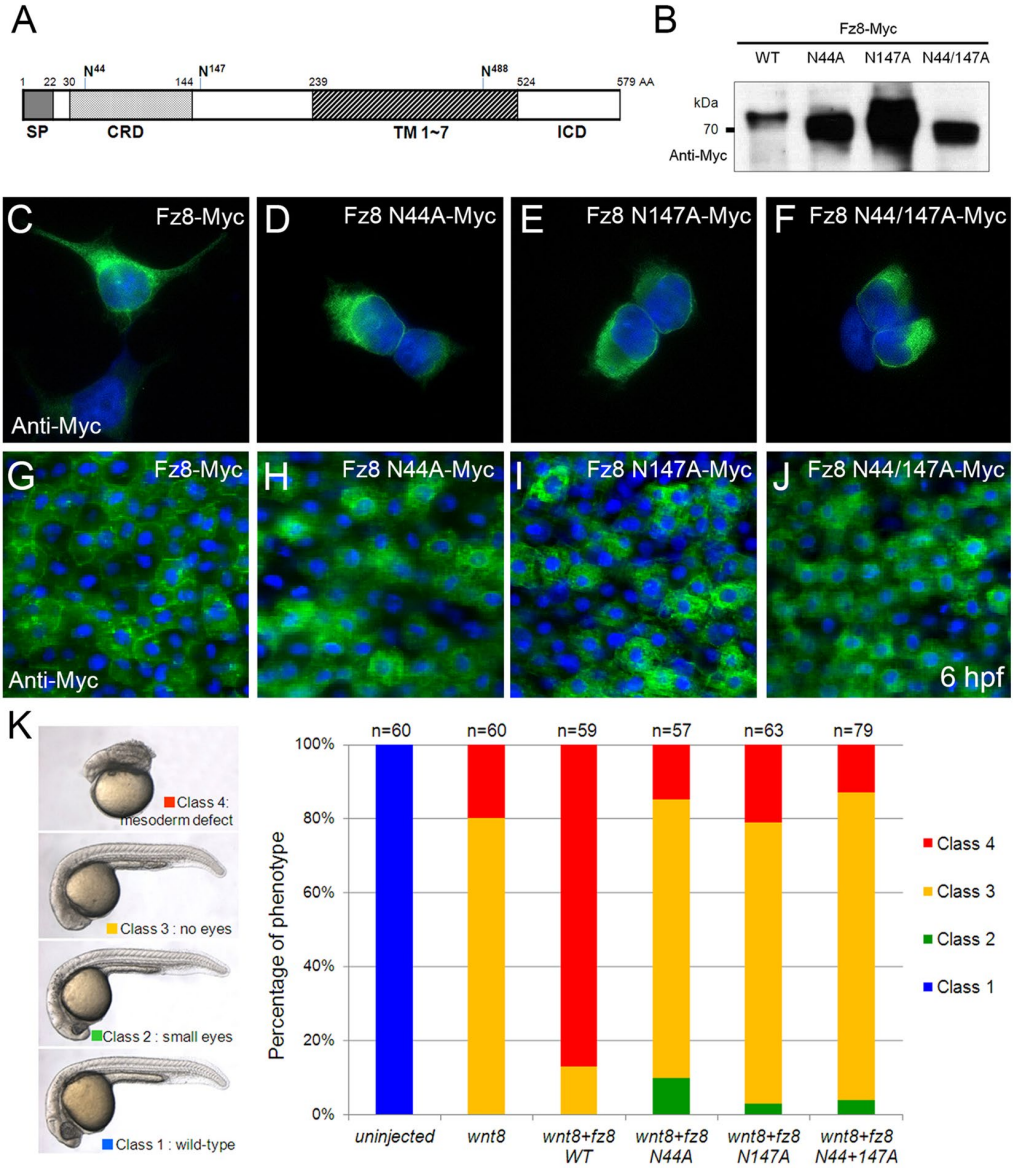
Putative N-glycosylation sites of Fz8 receptor are important for Wnt8 signaling. (**A**) Schematic representation of putative N-glycosylation sites in zebrafish Fz8a receptor. Abbreviations. CRD, Cystein rich domain; ICD, Intercellular domain; SP, Signal peptide; TM, Transmembrane domain. (**B–F**) HEK293 cells were transfected with indicated Fz8 construct and overexpressed Fz8 protein is analyzed by anti-Myc immunoblotting (**B**) or anti-Myc immunostaining (green) (**C**–**F**). For panels C–J, nuclei are counter-stained with Hoechst 33342 (blue). Full-length blots are presented in Supplementary Fig. S19. (**G—J**) Immunostaining of Myc (green) in embryos (6 hpf) injected with mRNA for indicated wild type or mutant *fz8a-myc*. (**K**) Embryos were injected with indicated combinations of *wnt8* and *fz8* mRNAs. Left panel: Resulting phenotypes are classified from Class I (normal) to Class 4 (most severe). Right panel: Phenotype frequencies of embryos injected with indicated combinations of mRNAs.

In order to assess potential roles of Fz8 glycosylation, we tested the activity of Fz8 mutants with Asn (N) to Ala (A) substitution at two putative N-glycosylation sites (Asn44 and Asn147) (Fig. 7A). When the proteins overexpressed in cultured cells were analyzed by SDS-PAGE, Fz8 N44A-Myc and Fz8 N44/147A-Myc mutants migrated slightly faster than did wild type Fz8-Myc (Fig. 7B). Then we examined subcellular localization of Fz8 mutant in cultured cells and zebrafish embryos. Unlike wild type Fz8-Myc, the majority of all Fz8 mutants demonstrated localization at the perinuclear region similar to wild type Fz8-Myc in TM-treated cells (Fig. 7C–J). These results demonstrate that glycosylation of Fz8 could be an essential factor in its membrane targeting.

Finally, we examined the effects of putative Fz8 glycosylation site mutants on Wnt signaling. Overexpression of Wnt8 caused either posteriorization (Class 3, 80%) or mesodermal defects (Class 4, 20%) in embryos (Fig. 7K). Co-injection of *wnt8* with *fz8* mRNA increased the proportion of more severe Class 4 phenotype (85%). However, co-injection of *fz8* mRNA encoding potential glycosylation-defective forms failed to enhance Wnt8-induced defects. Taken together, these results suggest that N-linked glycosylation of Fz8 may be critical for its cell surface localization and subsequently for Wnt/β-catenin signaling during early embryogenesis.

## Discussion

In this study, we identified *OTTOGI* (*OTG*), a novel negative regulator of Wnt signaling, by a large-scale expression screening of individual full-length human genes in zebrafish embryos. OTG overexpression caused inhibition of Wnt signaling whereas depletion of zebrafish *otg* caused aberrant activation of Wnt signaling in the various parameters that we examined. OTG interacted with Wnt receptor Fz8 and inhibited its cell surface targeting possibly by preventing N-linked glycosylation of Fz8.

The canonical Wnt/β-catenin signaling pathway is essential for specification of ventroposterior cell fates^30,31^. Results obtained from zebrafish studies have been consistent with a model derived from studies with *Xenopus*, wherein forebrain development is a default state and allowed only in the absence of BMP, Wnt, and Nodal signaling^32,33^. Studies have shown that Wnt activation leads to the suppression of anterior fates with concomitant expansion of posterior tissues. Conversely, inhibition of Wnt signaling has led to the expansion of anterior tissues at the expense of posterior domain^14,21,34^. OTG overexpression caused dorsoanteriorized phenotypes (Fig. 2) and suppressed the expression of direct Wnt target genes and TOPdGFP Wnt reporter (Fig. S7). However, *otg* depletion caused expanded the expression domains of ventroposterior markers at the expense of dorsoanterior markers and increased nuclear β-catenin accumulation (Fig. 4). As OTG mimics *in vivo* roles of previously reported Wnt inhibitors^35,36^, we concluded that OTG acts as a negative regulator of Wnt/β-catenin signaling during early embryonic development. Nevertheless, we cannot completely exclude the possibility that the *otg* depletion phenotype was not due to sequence-independent off-target effects of the morpholino; however, we did attempt to circumvent such non-specific phenotypes by suppressing p53 activity (Supplementary Fig. S6C) which can reduce ectopic cell death caused by non-specific morpholino effects. Thus, it is further necessary to use other more stable and reliable loss-of-function methods such as TALE nucleases and the Crispr/Cas9 system in future studies.

To test the genetic interaction between OTG and Wnt8, we simultaneously injected zebrafish embryos with *wnt8* and *otg* mRNAs (Fig. 3F). This double injection resulted in a phenotype similar to that of embryos injected with *otg* alone (Fig. 2C). This suggests that OTG can counter the activation of Wnt signaling by endogenous as well as exogenous Wnt ligands. However, the headless phenotype caused by inhibition of GSK3 (LiCl treatment) was not rescued by *otg* mRNA injection (Fig. 3I). These results suggest the possibility that OTG may function downstream to Wnt8 but upstream of GSK3. Apart from the head phenotype, it was not easy to explain the tail phenotype with this idea. Thus, we cannot exclude the possibility that OTG may have additional Wnt-independent functions during zebrafish embryogenesis.

We found that OTG could bind to the other Fzd2 and Fzd3 receptors, but this did not affect their subcellular distribution. However, OTG could both bind and affect the subcellular distribution of Fzd8, resulting in their specificity in AP-DV patterning. This indicates again that Fzd8 can uniquely control patterning with differential specificity compared to that of other Frizzled receptors. Fzd8 shows a very limited expression pattern in the specific region of the neuroectoderm at gastrulation stage. Thus, we can speculate that the tightly controlled expression pattern of Fzd8 and its binding to ubiquitous OTG are necessary for their unique function in patterning.

In *Xenopus*, Shisa transmembrane protein inhibits both Wnt and FGF signaling^7^. However, Shisa did not affect body patterning and cell surface expression of Fz8 in zebrafish embryos (data not shown). In contrast, Otg selectively inhibited membrane trafficking of Fz8 without affecting trafficking of FGF and BMP receptors (Supplementary Fig. S8I–L). Furthermore, OTG does not have any significant amino acid sequence homology to Shisa. Mesd/Boca, which acts as a chaperone of LRP6, promotes proper folding and cell surface localization of LRP6^37,38^. MG61/POPC, a *Drosophila* segment polarity gene product, stimulates N-glycosylation of Wnt ligands in the ER^39,40^. Unlike Mesd/Boca and MG61/POPC, Otg inhibits Fz8 maturation rather than promoting it. Glycosylation of Fz8 is critical for the onset of Wnt signaling by facilitating cell surface localization of Fz8^11^. We found that OTG binds to the N-terminal region of Fz8 which contains potential N-linked glycosylation sites. In addition, glycosylation defective forms of Fz8 were unable to translocate to the cell surface and failed to mediate Wnt8 signaling. OTG is localized in the ER but lacks a typical C-terminal ER retention signal (KDEL)^28^. Like OTG, Shisa^7^ and Wise^41^ also lack the KDEL sequence, but they sequester Fz8 and Lrp6 to the ER; respectively. As detailed molecular mechanisms of OTG function remain to be determined, it is intriguing to speculate whether OTG functions as a novel Wnt inhibitory scaffold protein in the recruitment of de-glycosylation enzymes and modulation of post-translation modification of Fz8 in the ER, in spite of its lack of discernible catalytic domains. Also, it is important to note that the antagonizing effect of OTG on Fz8 membrane targeting is not due to generic effects on membrane receptors, as per the findings that OTG does not affect membrane targeting of Fz10, Lrp5, Bmpr1 and Fgfr (Supplementary Fig. S8).

Wnt/β-catenin signaling is essential for kidney development. Accordingly, several Wnt family members are expressed in the embryonic kidney^42,43^. In mouse knock-out studies, Wnt9b-deficient mice showed defects in nephron induction^44^ while Wnt4-deficient mice exhibited renal agenesis due to defects in mesenchymal-to-epithelial transition^45^. Several Wnt signaling components have been shown to play important roles in pronephros development of *Xenopus* ^46^. *fz8a* transcript was detected in the pronephric duct during early pronephrogenesis in zebrafish (data not shown). We found that *otg* transcript is present in proximal convoluted tubules of the pronephros which is the most anterior region of the pronephric duct. Also, we could not identify any defects of pronephros in embryos where Otg activity was manipulated (data not shown), despite its restricted expression pattern.

Otg/Prr7 was first isolated as a protein which could interact and co-localize with the postsynaptic marker PSD-95 in rat hippocampal neurons^12^. PSD-95 is known to bind Fz receptors via C-terminus in cultured cells^47^. Wnt signaling acts as a key regulator in many aspects of synaptic organization and function^48,49^. Because of Otg interaction with Fz8 and inhibition of the Wnt pathway, our findings indicated that Otg plays an important role in synaptic formation and differentiation.

Recent bioinformatics searches characterized OTG/PRR7 as a transmembrane adaptor protein expressed in activated T cells. In Jurkat T cell line, overexpression of OTG/PRR7 caused caspase-dependent apoptosis^13^. Although cell- and tissue-specific effects of OTG/PRR7 cannot be ruled out, we did not see any significant apoptotic cell death in OTG-overexpressed zebrafish embryos and cultured cells (HEK293, COS-7, and NIH3T3 cells).

In conclusion, we utilized a large-scale expression screening to characterize a novel Wnt/β-catenin signaling inhibitor, OTG/PRR7. OTG controls D-V and A-P patterning during early embryonic development and prevents membrane targeting of Fz8 possibly by the inhibition of N-linked glycosylation. Given that aberrant regulation of Wnt signaling is frequently associated with various human degenerative diseases and cancers, investigating the roles of OTG may help to shed new light on the pathogenesis of such diseases.

## Materials and Methods

### Fish stocks and maintenance

Zebrafish was maintained at 28.5 °C under a 14 hr light, 10 hr dark cycle. Ages are given in hr post fertilization (hpf) based on standard developmental stages^50^. The *Tg[TOPdGFP]* transgenic fish was kindly provided by Dr. Richard Dorsky at University of Utah. Embryos older than 24 hpf were incubated with 0.003% 1-phenyl-2-thiourea (PTU) to inhibit pigmentation. All experimental protocols and procedures were approved and conducted according to the approved guidelines and regulations of the Animal Ethics Committee of Chungnam National University (CNU-00794).

### Large-scale expression screening of human genes

Human full-length cDNAs were provided by the Center for Functional Analysis of Human Genome at Korean Unigene Information (http://kugi.kribb.re.kr/). Methods for constructing the cDNA libraries are described elsewhere^51^. To make synthetic mRNA, each clone was linearized with appropriate restriction enzyme and *in vitro* transcribed with SP6, T3 or T7 mMessage Machine Kit (Ambion).

### Microinjection of synthetic mRNA, plasmid DNA, and morpholino

Synthetic mRNAs and plasmid DNAs were dissolved in 0.2 M KCl with 0.2% Phenol Red tracking dye, and microinjected into one to two cell stage embryos using a PV820 Pneumatic PicoPump (WPI). See Supplementary methods online for description of plasmids. Morpholino antisense oligonucleotides for *ottogi* 5′-AGG TGC CCT GCG ACA TCA CCA TGA T-3′, standard control 5′- CCT CTT ACC TCA GTT ACA ATT TAT A-3′, *p53* 5′-TTG ATT TTG CCG ACC TCC TCT CCA C-3′ were purchased from Gene-Tools, LLC. Each morpholino was resuspended in 1 × Danieau buffer (58 mM NaCl, 0.7 mM KCl, 0.4 mM MgSO_4_, 0.6 mM Ca(NO_3_)^2^, 5.0 mM HEPES pH 7.6) and injected into one- to two-cell stage embryos. The amount of mRNA and morpholinos is as follows: *wnt8* mRNA, 10 pg/embryo; *fz8*, *fz8* ^*N44A*^, *fz8* ^*N147A*^ mRNA, 50 pg/embryo; β*-catenin*, 100 pg/embryo; *VP16-tcf3* mRNA, 30 pg/embryo; *Myc-dvl1* mRNA, 50 pg/embryos; *fz10-Myc* mRNA, 50 pg/embryo, *bmpr1-Myc*, 10 pg/embryo; *otg*, *OTG*, *OTG-ER*, *OTG-TM*, *OTG-*Δ*TM*, *OTG-*Δ*PRD*, *OTG-*Δ*PBD* mRNA, 100 pg/embryo; *otg* MO; 10 ng/embryo; *wnt8* MO, 2 ng/embryo; *p53* MO, 2 ng/embryo. The final concentration of plasmid DNA including lrp5-HA, Fgfr-HA, OTG is 10 ng/ul.

### Whole-mount *in situ* hybridization and immunostaining

Whole-mount *in situ* hybridization was performed using digoxigenin-labeled RNA probes as previously described^52^. See Supplementary methods online for the list of probes used. Whole-mount immunostaining of zebrafish embryos was carried out as described previously^52^. For nuclei staining, immunostained zebrafish embryos and cultured cells were counter-stained with Hoechst 33342 at 1 mg/ml and 10 μg/ml, respectively. To photograph the blastula or gastrula stage embryo, the yolk sac was removed and the remaining embryo was laid flat on slide glass. Embryo preparations were examined under a confocal microscope.

### Cell culture and transient transfection

Cells were cultured in DMEM with 10% fetal calf serum. Transfection was performed using Lipofectamine Plus reagent (Invitrogen) according to the manufacturer’s instruction.

### Immunoprecipitation and immunoblotting

Transfected cells were lysed in an ice-cold lysis buffer (20 mM Hepes pH 7.9, 300 mM NaCl, 100 mM KCl, 10 mM EDTA, 0.2% NP-40, and 10% glycerol) with protease inhibitor cocktail (Roche Applied Science). Cell lysates were immunoprecipitated with protein A-agarose bead conjugated anti-FLAG or anti-Myc antibody. Immunoprecipitated proteins and total cell lysates were subjected to SDS-PAGE and transferred to nitrocellulose membrane. Proteins were visualized by ECL substrate (Thermo Scientific).

### Chemical treatment and enzymatic reaction

Zebrafish gastrulae (6 hpf) were treated with lithium chloride (0.3 M in egg water) and SB415286 (20 mM in DMSO) for 15–20 min and with tunicamycin (3 μg/ml) for 4 hr at 28.5 °C. Transfected cells were treated with tunicamycin for 24 hr. For endoglycosidase reaction, cell lysates were incubated with Endo H or O-glycosidase (ElpisBiotech) for 2 hr at 37 °C.

### In brief

We isolated a novel Wnt inhibitor, OTG, from a large-scale expression screening of human cDNAs in zebrafish. Overexpression of OTG inhibited Wnt-induced responses, but knockdown of OTG enhanced Wnt-induced phenotypes. OTG bound specifically to Frizzled8 (Fz8) receptor and caused retention of Fz8 in the endoplasmic reticulum.

## Electronic supplementary material

Supplementary information
