# Supplementary material for: Ottogi Inhibits Wnt/β-catenin Signaling by Regulating Cell Membrane Trafficking of Frizzled8
Source: Sci Rep. 2017 Oct 16;7:13278. doi: 10.1038/s41598-017-13429-6 (PMC5643531; doi:10.1038/s41598-017-13429-6)
Supplement: Supplementary file 1 — Supplementary information [file 41598_2017_13429_MOESM1_ESM.pdf]

Supplementary Information

Ottogi Inhibits Wnt/β-catenin Signaling by Regulating Cell Membrane Trafficking of Frizzled8

Hyun-Taek Kim, Mi-Sun Lee, Yun-Mi Jeong, Hyunju Ro, Dong-Il Kim, Yong-Hwan Shin, Ji-Eun Kim, Kyu-Seok Hwang, Jung-Hwa Choi, Minjin Bahn, Jeong-Ju Lee, Sang H. Lee, Young-Ki Bae, Jin-Soo Lee, Joong-Kook Choi, Chang-Yeol Yeo, Nam-Soon Kim, and Cheol-Hee Kim

Supplementary Experimental Procedures

Plasmids

Zebrafish *ottogi* gene was isolated from 24 hpf zebrafish cDNA library, cloned into pGEM-T easy vector and then subcloned into EcoRI site in pCS2+ vector. Plasmids containing full-length ORF or truncated forms of human OTTOGI or zebrafish ottogi were constructed in pCS2+EGFP or pCS2+FLAG vector by PCR-based methods. Zebrafish *frizzled8a*, *frizzled8b*, *frizzled10*, and *bmpr1a* genes were isolated from the 24 hpf zebrafish cDNA library and subcloned to pCS2+MT. The *fz8aΔC*, *fz8aΔN*, *fz8aΔCRD*, *fz8a N44A*, *fz8a N147A*, *fz8a N44A/N147A* constructs were made in pCS2+MT vector by PCR-based methods. *Xenopus fgfr-HA* and *lrp6-HA* were kindly provided from Dr. Shinichi Aizawa (RIKEN, Japan).

Whole-mount *in situ* hybridization and paraffin section

Antisense digoxigenin-labeled RNA probes for *pax6* (Macdonald et al., 1994), *chd* (Miller-Bertoglio et al., 1997), *gsc* (Stachel et al., 1993), *otx2* (Mori et al., 1994), *bmp2* (Nguyen et

al., 1998), *eve1* (Joly et al., 1993), *hoxa1* (Alexandre et al., 1996), *dha (*Koos & Ho, 1998), *gata1* (Gering et al., 1998), *six3 (*Kobayashi et al., 1998), *spt/tbx16* (Amacher & Kimmel, 1998), *tbx6* (Hug et al., 1997), *tlc/sfrp5* (Houart et al., 2002), *sox2* (Dee et al., 2008), *wnt11* (Makita et al., 1998), *mixer/bon* (Alexander & Stainier, 1999), *shh* (Krauss et al., 1993), *zic1* (Grinblat et al., 1998), *rx1* (Rojas- Muñoz et al., 2005), *krox20* (Strähle et al., 1993), *otg*, and *gfp* were synthesized using DIG-RNA labeling kit (Roche Applied Science) according to the manufacturer's instruction. Whole-mount *in situ* hybridization was performed as previously described (Kim et al., 2008). For paraffin embedding, embryos were dehydrated with ethanol for 5 min and then cleared three times for 10 min in xylene. Specimens in xylene were embedded in paraffin and cut at 6 μm thickness.

Membrane topology of OTG protein

For permeabilized immunofluorescence, cultured cells were fixed with 4 % paraformaldehyde in PBS for 15 min and then permeabilized in 2 % BSA/0.05 % saponin/PBS for 30 min at room temperature. For intact cell immunofluorescence, cells were washed with ice cold PBS/BSA solution and then incubated with primary antibodies on ice for 1 h in ice cold BSA/PBS. The experiments were done with human PRR7.

Supplementary References

Alexander, J., and Stainier, D.Y. (1999). A molecular pathway leading to endoderm formation in zebrafish. Curr. Biol. *9*, 1147-1157.

Alexandre, D., Clarke, J.D., Oxtoby, E., Yan, Y.L., Jowett, T., and Holder, N. (1996). Ectopic expression of *Hoxa-1* in the zebrafish alters the fate of the mandibular arch neural crest and phenocopies a retinoic acid-induced phenotype. Development *122*, 735-746.

Amacher, S.L., and Kimmel, C.B. (1998). Promoting notochord fate and repressing muscle development in zebrafish axial mesoderm. Development *125*, 1397-1406

Dee, C.T., Hirst, C.S., Shih, Y.H., Tripathi, V.B., Patient, R.K., and Scotting, P.J. (2008). *Sox3* regulates both neural fate and differentiation in the zebrafish ectoderm. Dev. Biol. *320*, 289-301.

Gering, M., Rodaway, A.R., Göttgens, B., Patient, R.K., and Green, A.R. (1998). The *SCL* gene specifies haemangioblast development from early mesoderm. EMBO J. *17*, 4029-4045.

Grinblat, Y., Gamse, J., Patel, M., and Sive, H. (1998). Determination of the zebrafish forebrain: induction and patterning. Development *125*, 4403-4416.

Hug, B., Walter, V., and Grunwald, D.J. (1997). *tbx6*, a *Brachyury*-related gene expressed by ventral mesendodermal precursors in the zebrafish embryo. Dev. Biol. *183*, 61-73.

Houart, C., Caneparo, L., Heisenberg, C., Barth, K., Take-Uchi, M., and Wilson S. (2002). Establishment of the telencephalon during gastrulation by local antagonism of Wnt signaling. Neuron *35*, 255-265.

Joly, J.S., Joly, C., Schulte-Merker, S., Boulekbache, H. and Condamine, H. (1993). The ventral and posterior expression of the zebrafish homeobox gene *eve1* is perturbed in dorsalized and mutant embryos. Development *119*, 1261-1275.

Krauss, S., Concordet, J.P., and Ingham, P.W. (1993). A functionally conserved homolog of the Drosophila segment polarity gene *hh* is expressed in tissues with polarizing activity in zebrafish embryos. Cell *75*, 1431-1444.

Kim, H.T., Kim, E.H., Yoo, K.W., Lee, M.S., Choi, J.H., Park, H.C., Yeo, S.Y., Lee, D.S., and Kim, C.H. (2008). Isolation and expression analysis of Alzheimer's disease-related gene *xb51* in zebrafish. Dev. Dyn. *237*, 3921-3926.

Kobayashi, M., Toyama, R., Takeda, H., Dawid, I.B. and Kawakami, K. (1998). Overexpression of the forebrain specific homeobox gene *six3* induces rostral forebrain enlargement in zebrafish. Development *125*, 2973-2982.

Koos, D.S., and Ho, R.K. (1998). The *nieuwkoid* gene characterizes and mediates a Nieuwkoop-center-like activity in the zebrafish. Curr. Biol. *8*, 1199-1206.

Macdonald, R., Xu, Q., Barth, K.A., Mikkola, I., Holder, N., Fjose, A., Krauss, S., and Wilson, S.W. (1994). Regulatory gene expression boundaries demarcate sites of neuronal differentiation in the embryonic zebrafish forebrain. Neuron *13*, 1039-1053.

Makita, R., Mizuno, T., Koshida, S., Kuroiwa, A., and Takeda, H. (1998). Zebrafish *wnt11*: pattern and regulation of the expression by the yolk cell and No tail activity. Mech. Dev. *71*, 165-176.

Miller-Bertoglio, V.E., Fisher, S., Sanchez, A., Mullins, M.C., and Halpern M.E. (1997). Differential regulation of *chordin* expression domains in mutant zebrafish. Dev. Biol. *192*, 537-550.

Mori, H., Miyazaki, Y., Morita, T., Nitta, H., and Mishina, M. (1994). Different spatio-temporal expressions of three *otx* homeoprotein transcripts during zebrafish embryogenesis. Brain Res. Mol. Brain Res. *27*, 221-231.

Nguyen, V.H., Schmid, B., Trout, J., Conners, S.A., Ekker, M., and Mullins, M.C. (1998). Ventral and lateral regions of the zebrafish gastrula, including the neural crest progenitors, are established by a *bmp2b*/*swirl* pathway of genes. Dev. Biol. *199*, 93-110.

Rojas-Muñoz, A., Dahm, R., and Nüsslein-Volhard, C. (2005). *chokh*/*rx3* specifies the retinal pigment epithelium fate independently of eye morphogenesis. Dev. Biol. *288*, 348-362.

Stachel, S. E., Grunwald, D. J., and Myers, P. Z. (1993). Lithium perturbation and *goosecoid* expression identify a dorsal specification pathway in the pregastrula zebrafish. Development *117*, 1261-1274.

Strähle, U., Blader, P., Henrique, D., and Ingham P.W. (1993). *Axial*, a zebrafish gene expressed along the developing body axis, shows altered expression in *cyclops* mutant embryos. Genes Dev. *7*, 1436-1446.

Supplementary table. Summary of injection experiment values.

**Fig. 2**	chd		gsc		bmp2		eve1		otx2/hoxa1		pax6	
	F	20/20, 100%	H	15/15, 100%	J	12/12, 100%	L	10/10, 100%	N	12/12, 100%	P	20/20, 100%
	G	13/15, 87%	I	14/15, 93%	K	11/12, 92%	M	11/12, 92%	O	15/16, 94%	Q	14/16, 87%
**Fig. 3**	dha		wnt8 - 30hpf		LiCl - 24 hpf		tbx6		dkk1			
	A	10/10, 100%	D	10/10, 100%	G	12/12, 100%	J	10/10, 100%	L	10/10, 100%		
	B	14/15, 93%	E	18/20, 90%	H	13/15, 86%	K	18/20, 90%	M	17/20, 85%		
	C	12/13, 92%	F	17/20, 85%	I	14/15, 93%						
**Fig. 4**	six3 + hoxa1		spt									
	A	12/12, 100%	D	10/10, 100%								
	B	17/20, 85%	E	18/20, 90%								
	C	16/18, 89%	F	20/23, 87%								
**Fig. S3**	bmp4		tlc/sfrp5		sox2		wnt11		mixer			
	A	10/10, 100%	C	12/12, 100%	E	10/10, 100%	G	11/11, 100%	I	10/10, 100%		
	B	14/15, 93%	D	13/15, 86%	F	12/13, 92%	H	12/12, 100%	J	10/10, 100%		
**Fig. S4**	chd		b-catenin inj		vp16-tcf3 inj		SB415286					
	A	10/10, 100%	D	10/10, 100%	G	10/10, 100%	J	10/10, 100%				
	B	8/10, 80%	E	12/15, 80%	H	14/18, 78%	K	14/15, 93%				
	C	10/12, 83%	F	13/14, 93%	I	16/18, 89%	I	14/18, 78%				
**Fig. S6**	24 hpf		4 ss		30 hpf		zic/opl		rx1+kx20			
	A	20/20, 100%	D	10/10, 100%	G	15/15, 100%	J	14/14, 100%	M	12/12, 100%		
	B	23/25, 92%	E	14/15, 93%	H	14/16, 87%	K	20/22, 91%	N	15/16, 94%		
	C	22/23, 96%	F	13/13, 100%	I	10/15, 66%	L	18/20, 90%	O	20/22, 91%		
**Fig. S7**	A	10/10, 100%	B	12/13, 92%	C	12/14, 86%	D	18/20, 90%				
	E	7/7, 100%	F	8/10, 80%	G	6/10, 60%	H	14/16, 87%				
**Fig. S10**	A	13/13, 100%	B	20/21, 95%	C	22/24, 92%						
	D	10/10, 100%	E	12/13, 92%	F	11/13, 85%						
**Fig. S15**	A	12/12, 100%	B	15/15, 100%	C	10/14, 71%	D	11/15, 73%	E	12/16, 75%	F	15/16, 94%

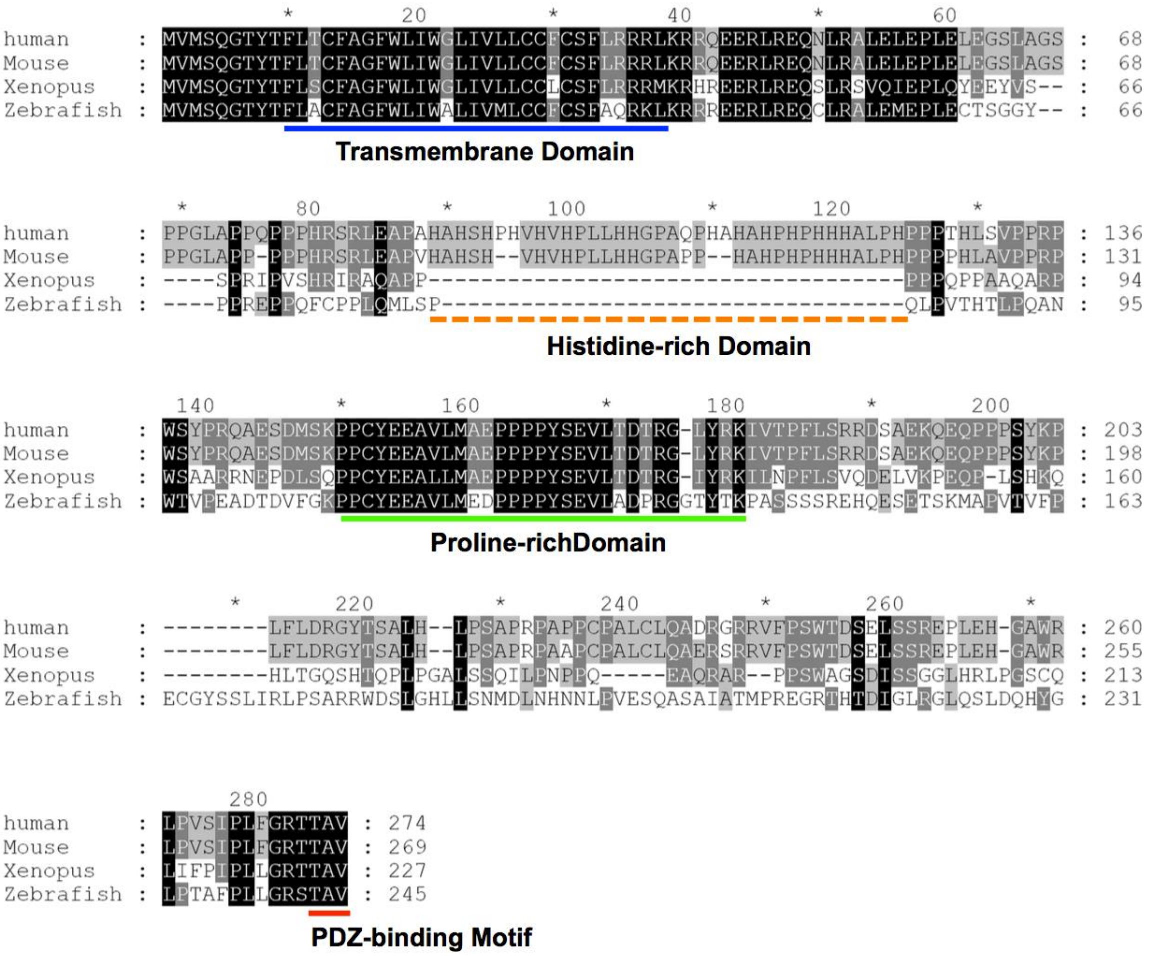

Supplementary information, Figure S1. Amino acid sequence comparison of vertebrate OTG homologues

Zebrafish *otg* gene encodes a 245 amino acids-long polypeptide containing a transmembrane domain (blue underline), a proline-rich domain (green underline), and a C-terminal PDZ-binding motif (red underline). Amino acid sequence of the zebrafish Otg displays 48% and 51% similarity to human/mouse and *Xenopus* homologues, respectively. Zebrafish and *Xenopus* Otg lack a histidine-rich domain (red dotted underline) present in human and mouse homologues.

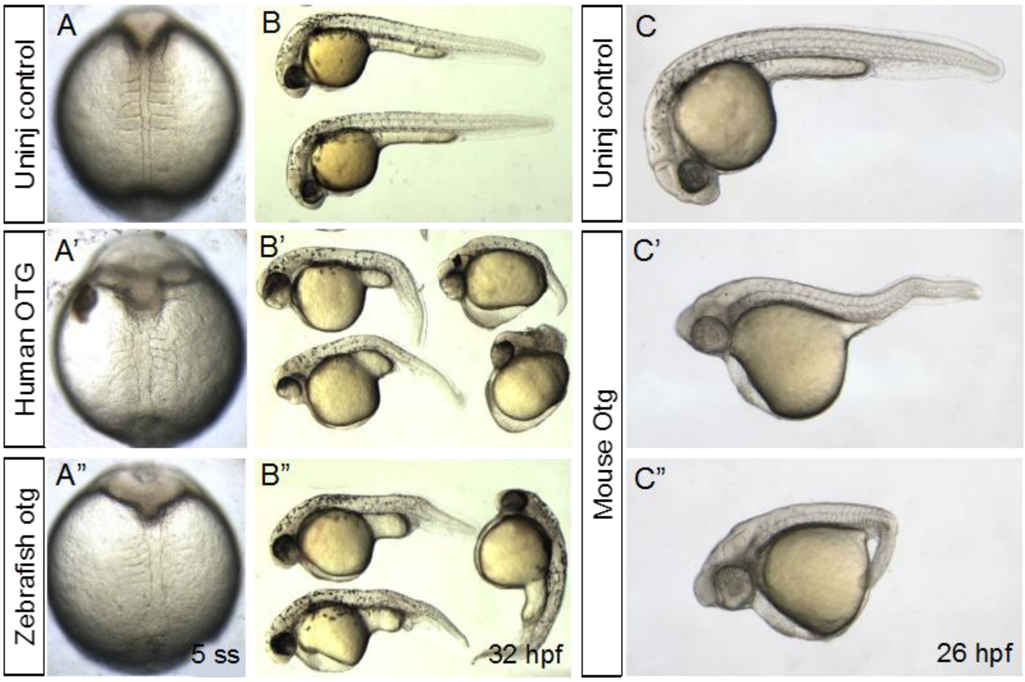

Supplementary information, Figure S2. Overexpression analysis of human, mouse, and zebrafish OTG genes

(A-A”) Dorsal views of five-somite stage embryos injected human (A’) and zebrafish OTG (A”). Enlargement of head region is prominent both in human and zebrafish OTG injected embryos, compared to that of control embryo. Dorsal view, anterior is to the top.

(B-B”) Lateral views of 32 hpf embryos injected human (B’) and zebrafish OTG (B”).

(C-C”) Big head phenotype is also seen in mouse Otg injected embryos at 26 hpf.

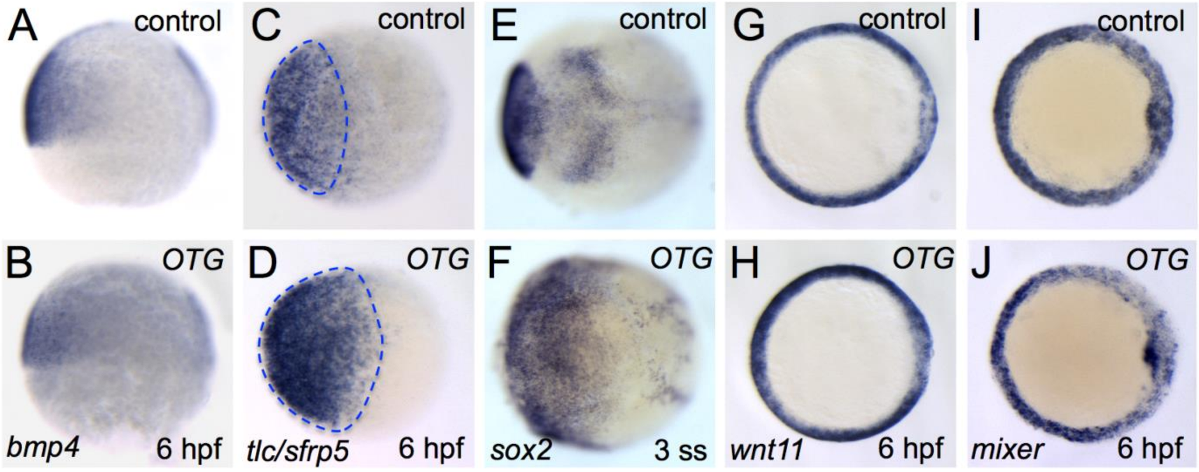

Supplementary information, Figure S3. Expression patterns of early markers in OTG-overexpressed embryos

Expression of patterns of a ventral marker *bmp4* (A, 10/10, 100%; B, 14/15, 93%), an anterior neural plate marker *tlc/sfrp5* (C, 12/12, 100%; D, 13/15, 86%), a pan-neural marker *sox2* (E, 10/10, 100%; F, 12/13, 92%), endoderm markers *wnt11* (G, 11/11, 100%; H, 12/12, 100%) and *mixer* (I, 10/10, 100%; J, 10/10, 100%) in un-injected control (A, C, E, G, I) or *OTG* mRNA-injected (B, D, F, H, J) embryos. (C, D and G-J) Embryos at the onset of gastrulation (6 hpf), views from animal pole, dorsal on the right side. (A, B) Side view. (E-F) Embryos at 3 somite stage (3 ss), dorsal views, anterior to the left. Note that the expression domains of *bmp4*, *tlc/sfrp5* and *sox2* are expanded in OTG-overexpressed embryos whereas those of *wnt11* and *mixer* are not affected.

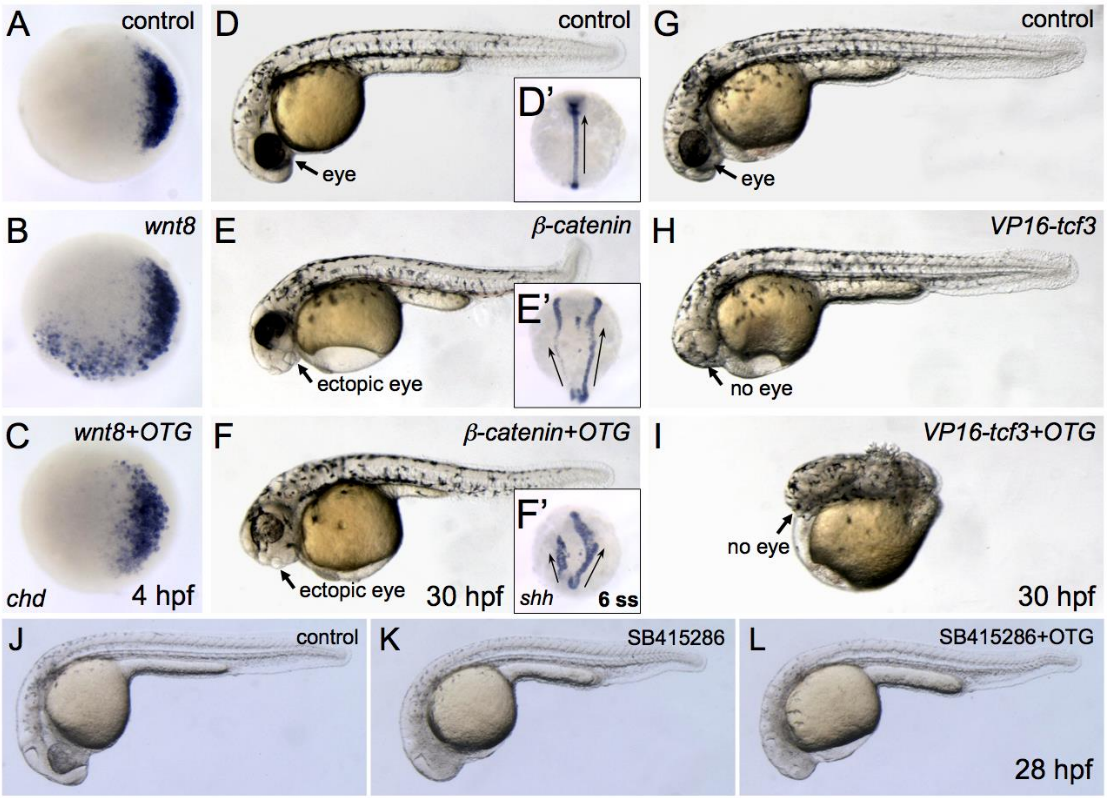

Supplementary information, Figure S4. OTG acts upstream of *β-catenin* and *tcf3/hdl* in Wnt signaling pathway

(A-C) Top view, dorsal is to the right. Expression pattern of *chd* in control (A, 10/10, 100%), *wnt8* mRNA-injected (B, 8/10, 80%), or *wnt8* and *OTG* mRNA-injected (C, 10/12, 83%) embryo at sphere stage (4 hpf). Wnt8-induced ectopic expression of *chd* was inhibited by OTG overexpression.

(D-F') Morphology and expression pattern of *shh* (insets) in control (D, 10/10, 100%; D'), *β-catenin* mRNA-injected (E, 12/15, 80%; E'), or *β-catenin* and *OTG* mRNA-injected (F, 13/14, 93%; F') embryo at 30 hpf and 6 ss. OTG overexpression does not inhibit β-catenin-induced ectopic eye formation or secondary axis formation marked by the ectopic expression of *shh*.

(G-I) Morphology of control (G, 10/10, 100%), *VP16-tcf3* (a dominant negative form of tcf3) mRNA-injected (H, 14/18, 78%), or *VP16-tcf3* and *OTG* mRNA-injected (I, 16/18, 89%) embryo at 30 hpf. OTG overexpression does not rescue VP16-tcf3-induced *headless*-like phenotype.

(J-L) Morphology of control (J, 10/10, 100%), SB415286 treated (K, 14/15, 93%), or SB415286 treated and *OTG* mRNA-injected embryo (L, 14/18, 78%) at 28 hpf. Injection of *OTG* mRNA does not rescue SB415286-induced headless-like phenotype.

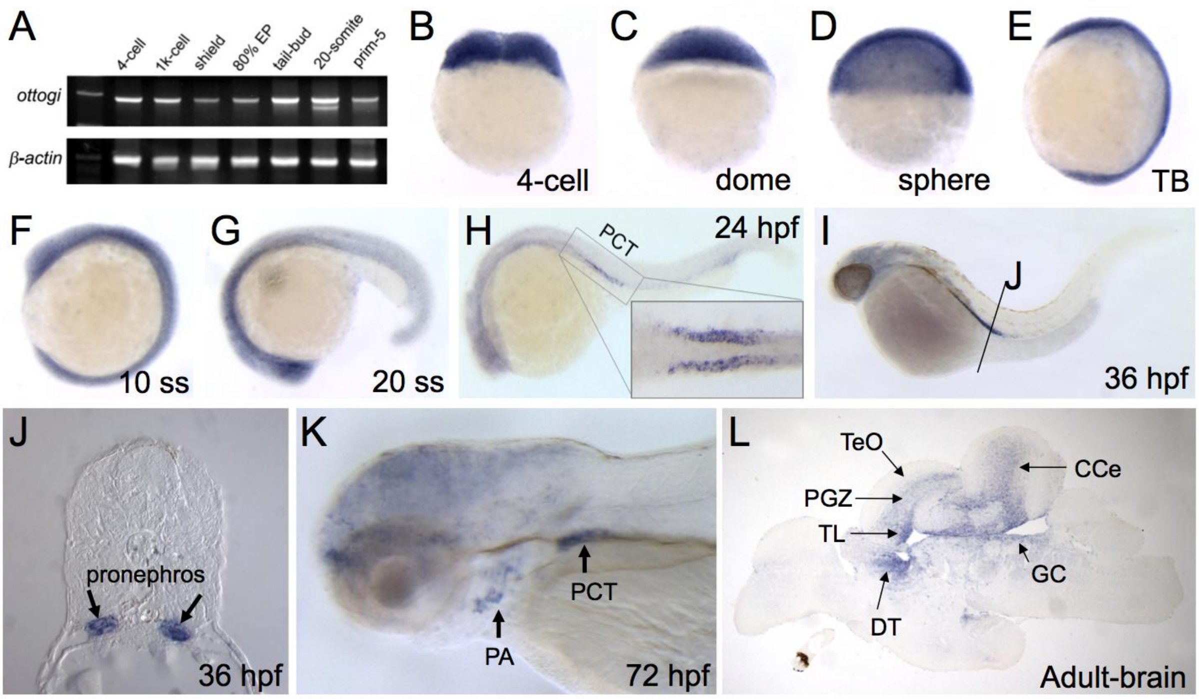

Supplementary information, Figure S5. Expression profile of *otg* in zebrafish embryos

(A) Semi-quantitative RT-PCR analysis of zebrafish *ottogi*. *β-actin* is used as a loading control. *ottogi* transcript is detected from 4-cell to the prim-5 stages (24 hpf).

(B-L) *In situ* hybridization of zebrafish *otg* at indicated stages. (B-F) *otg* is ubiquitously expressed from early cleavage to 10-somite stages. (G) *otg* expression in brain region is increased at late somitogenesis stage. (H) At 24 hpf, *otg* transcripts are detected in the brain and proximal convoluted tubule (PCT, inset) of pronephros. (I) At 36 hpf, *otg* expression persists in the brain and pronephric ducts. (J) Cross section of embryo at panel (I) shows that *otg* transcripts are detected in anterior pronephric ducts (arrows) at 36 hpf. (K) *otg* transcripts are detected in the brain, pharyngeal arch (PA), and PCT at 72 hpf. (L) In sagittal section of adult brain, *otg* transcripts are detected in corpus cerebelli (CCe), dorsal thalamus (DT), griseum centrale (GC), periventricular gray zone of optic tectum (PGZ, layers 1 and 2), and torus longitudinalis (TL). TeO indicates tectum opticum.

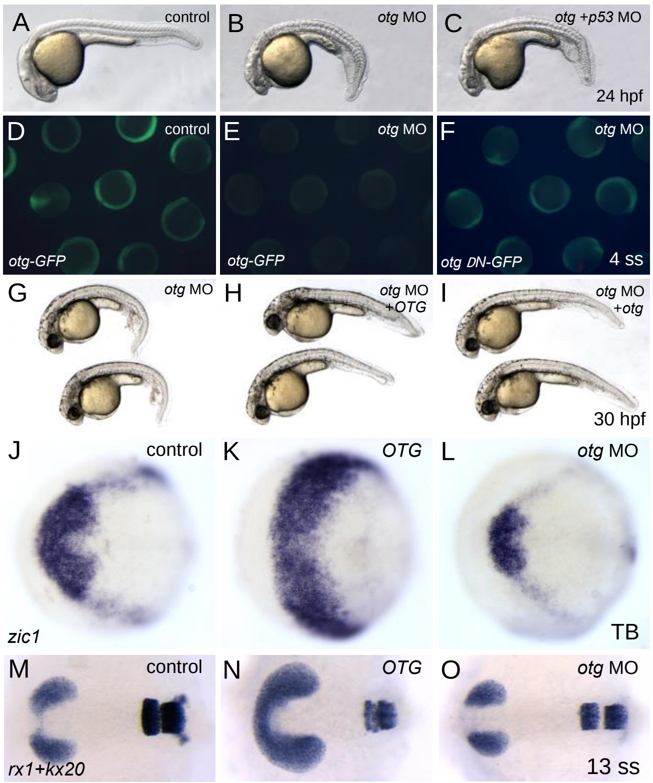

Supplementary information, Figure S6. Loss of *otg* function leads to a ventralized phenotype.

(A-C) Morphology of control (A, 20/20, 100%), *otg* MO-injected (B, 23/25, 92%), or *otg* and *p53* MO-injected (C, 22/23, 96%) embryo at 24 hpf. Injection of *otg* MO causes a ventralized phenotype (including small head/eye and expansion of ventral mesoderm) and co-injection of *p53* MO does not affect the ventralized phenotype of *otg* morphants. (D-F) GFP expression at 4-somite stage embryos injected with indicated combinations of control MO, *otg* MO, *otg-GFP* (otg MO-targeted) mRNA and *otg-ΔN-GFP* (otg MO non-targeted) mRNA. Otg-GFP is robustly expressed in control MO-injected embryos (D, 10/10, 100%) but not in *otg* MO-injected embryos (E, 14/15, 93%). Expression of Otg-ΔN-GFP is not affected by *otg* MO (F, 13/13, 100%). (G-I) Morphology of 30 hpf embryos injected with *otg* MO alone (G, 15/15, 100%), or with human *OTG* mRNA (H, 14/16, 87%) or zebrafish *otg* mRNA (I, 10/15, 66%). *otg* MO-induced ventralized phenotype (including expansion of ICM and small head) is rescued by *OTG*/*otg* mRNA co-injection. (J-L) Expression pattern of a presumptive eye field marker *zic1/opl* in control (J, 14/14, 100%), *OTG* mRNA-injected (K, 20/22, 91%) or *otg* MO-injected (L, 18/20, 90%) embryo at neural plate stage. Dorsal view, anterior is to the left. Overexpression of OTG caused dramatic expansion of *zic1/opl* expression domain whereas loss of *otg* function caused reduction. (M-O) Expression pattern of an eye field marker *rx1* and a hindbrain markers *krox20* in control (M, 12/12, 100%), *OTG* mRNA-injected (N, 15/16, 94%) or *otg* MO-injected (O, 20/22, 91%) embryo at 13-somite stage. Dorsal view, anterior is to the left. Overexpression of OTG caused expansion of *rx1* expression domain whereas loss of *otg* function caused reduction.

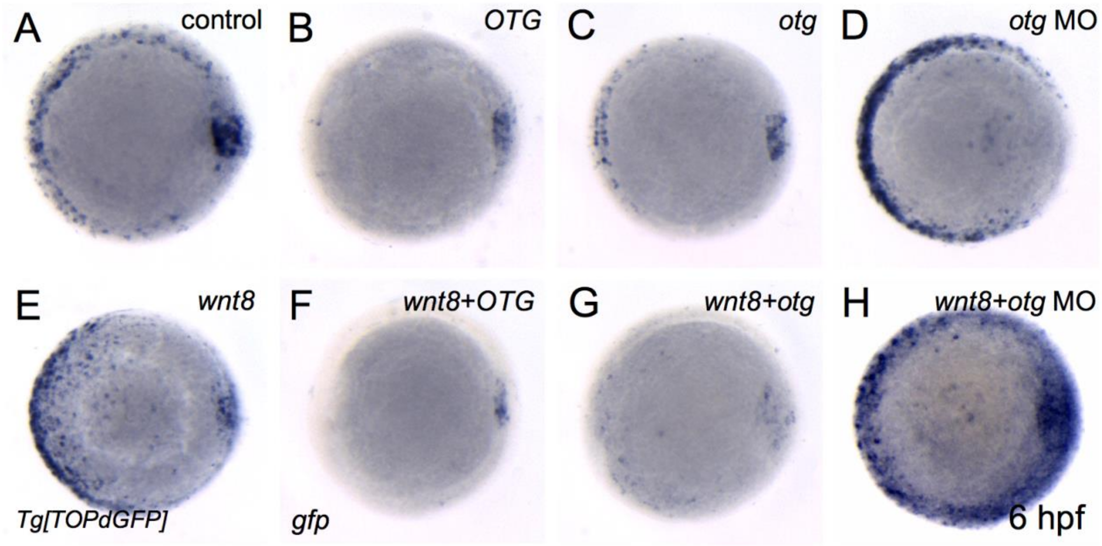

Supplementary information, Figure S7. OTG inhibits reporter expression in TOPdGFP transgenic zebrafish

Views from animal pole, dorsal is to the right. Patterns of GFP reporter expression in control (A, 10/10, 100%), human *OTG* mRNA-injected (B, 12/13, 92%), zebrafish *otg* mRNA-injected (C, 12/14, 86%), or *otg* MO-injected (D, 18/20, 90%) embryo at shield stage (6 hpf). E-H. OTG overexpression suppresses whereas otg knockdown enhances Wnt8-induced reporter expression. *TOPdGFP* transgenic embryos were injected with indicated combination of *wnt8* (7/7, 100%), human *OTG* (8/10, 80%), zebrafish *otg* mRNAs (6/10, 60%) and zebrafish *otg* morpholino (MO, 14/16, 87%). GFP reporter expression is examined by in situ hybridization.

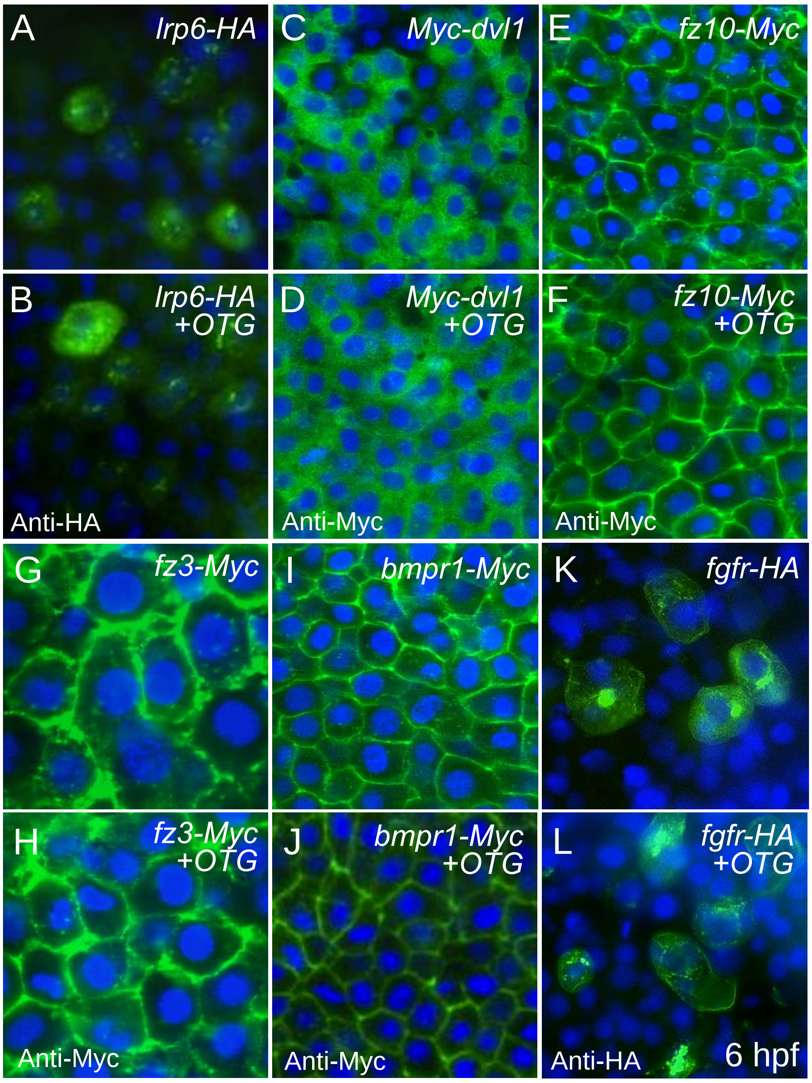

Supplementary information, Figure S8. OTG does not affect subcellular localization of Lrp6, Dvl1, Fz3, Fz10, Bmpr1, and Fgfr

(A-J) Immunostaining of gastrula embryos (6 hpf) injected with indicated combinations (top right corner) of mRNA. Subcellular localization of overexpressed proteins are examined by anti-HA (A-B, K,L) or anti-Myc (C-J) immunostaining. Nuclei are counter-stained with Hoechst 33342 (blue). OTG overexpression does not affect the subcellular localization of Lrp6-HA (A, B), Myc-Dvl1 (C, D), Fz10-Myc (E-F), Fz3-Myc (G, H), Bmpr1-Myc (I, J), or Fgfr-HA (K, L) in zebrafish embryo.

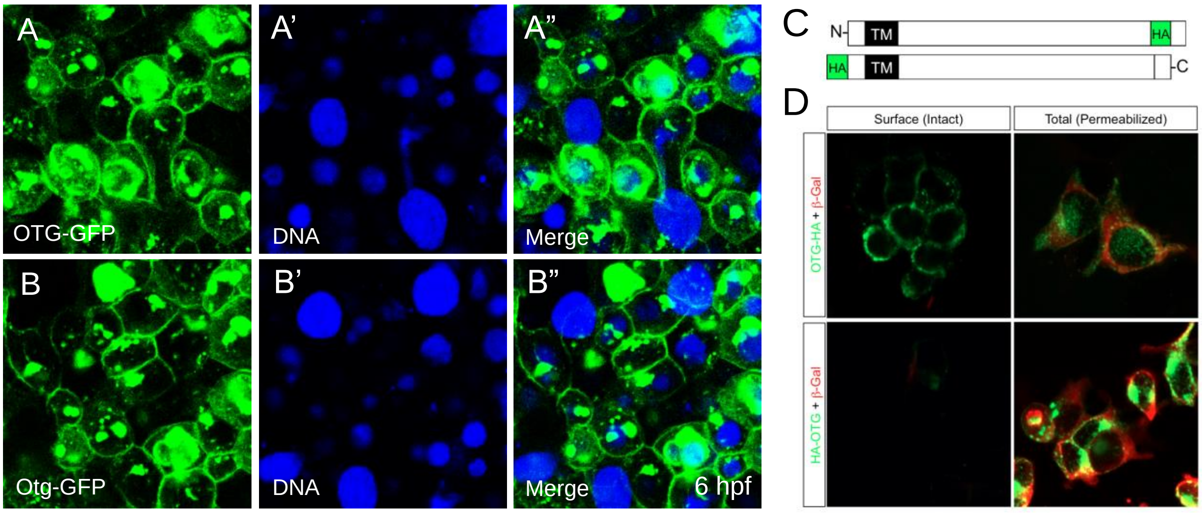

Supplementary information, Figure S9. OTG is distributed in perinuclear sites and the plasma membrane

(A-B'') Immunostaining of gastrula embryos injected with human *OTG-GFP* (A-A'') or zebrafish *otg-GFP* (B-B'') mRNA. Nuclei are counter-stained with Hoechst 33342 (blue). Human OTG-GFP and zebrafish Otg-GFP are mainly localized in perinuclear sites and the plasma membrane. (C,D) Membrane topology assay of OTG proetins. C. Schematic representation of differentially HA epitope-tagged OTG proteins (OTG-HA and HA-OTG, respectively). D. OTG-HA or HA-OTG proteins were transiently expressed in the HEK293 cells. In intact cell immunofluorescence, the HA epitope was detected in the cell surface of OTG-HA transfected cells, but it was hardly detected in HA-OTG transfected cells. However, in permeabilized immunofluorescence, the HA epitope was detected in both OTG-HA and HA-OTG cells. In the absence of membrane permeabilization, HA epitope was detected at the cell surface only in OTG-HA transfected cells but not in HA-OTG transfected cells. However, after detergent (Triton X-100) treatment of cells, HA was detected in both OTG-HA and HA-OTG cells.

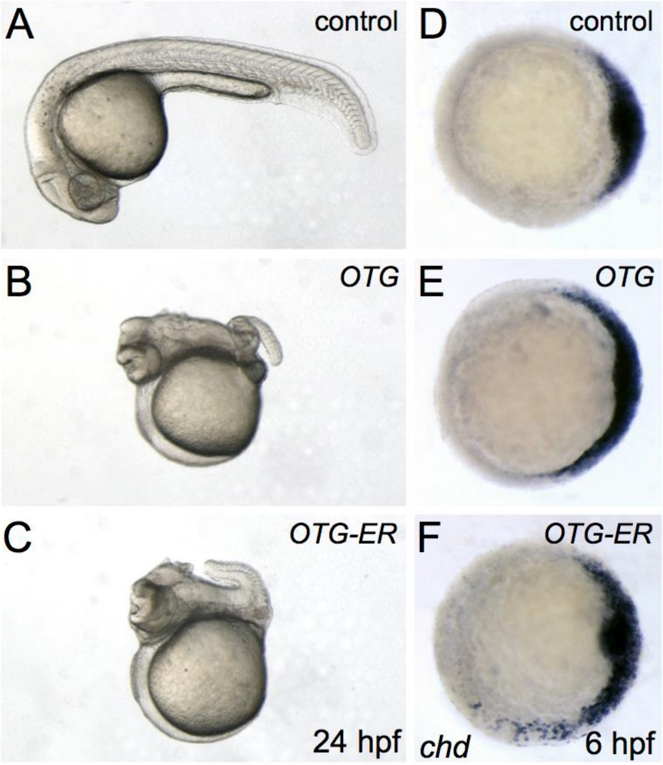

Supplementary information, Figure S10. ER-restricted OTG (Otg-ER) retains its activity

(A-F) Morphology (A-C, at 24 hpf) and expression pattern of a dorsal organizer marker *chd* (D-F, 6 hpf; animal pole view, dorsal is to the right) of control (A, 13/13, 100%; D, 10/10, 100%), *OTG* mRNA-injected (B, 20/21, 95%; E, 12/13, 92%), or an ER-restricted *OTG-ER* mRNA-injected (C, 22/24, 92%; F, 11/13, 85%) embryos. Similar to wild type OTG, OTG-ER causes dorsalization and expansion of *chd* expression domain.

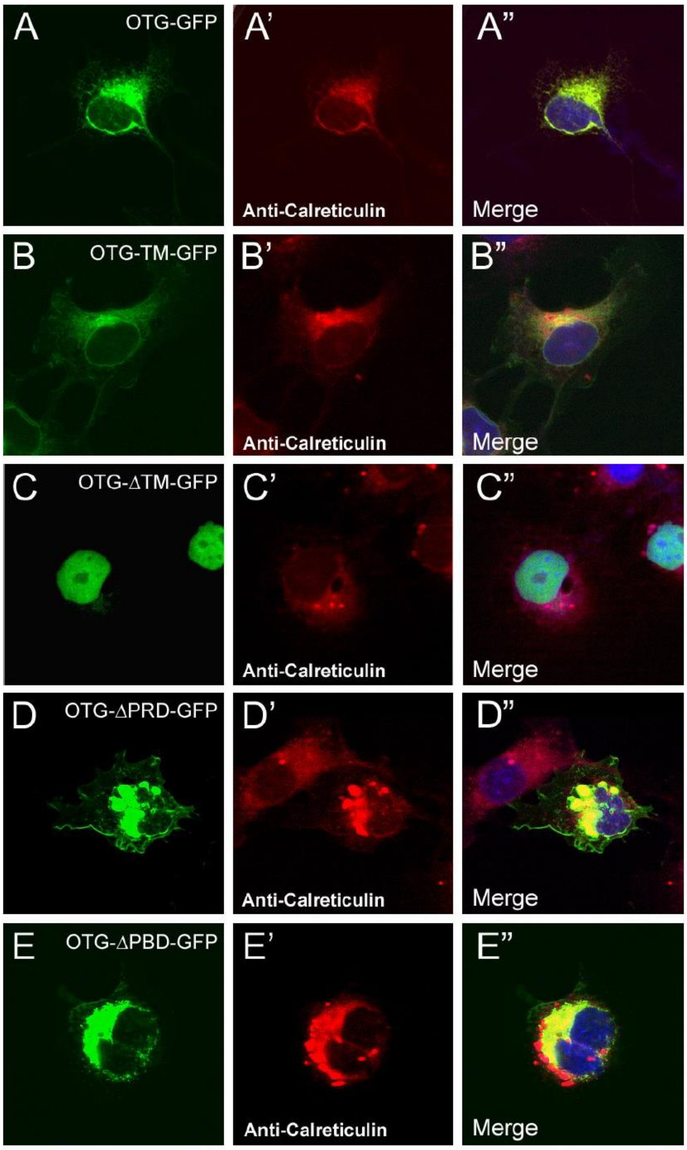

Supplementary information, Figure S11. ER localization of OTG requires its amino-terminal region

(A-E'') Immunostaining of COS-7 cells transfected with OTG wild type or various deletion constructs. Overexpressed OTG proteins are visualized by anti-GFP immunostaining (green) along with calreticulin (red). Nuclei are counter-stained with Hoechst 33342 (blue). OTG TM-GFP (B-B"), OTG ΔPRD-GFP (D-D"), and OTGΔPBD-GFP (E-E") are localized in similar patterns as wild type OTG-GFP. However, OTG ΔTM-GFP, lacking the transmembrane domain located near N-terminus, is localized in the nucleus (C-C").

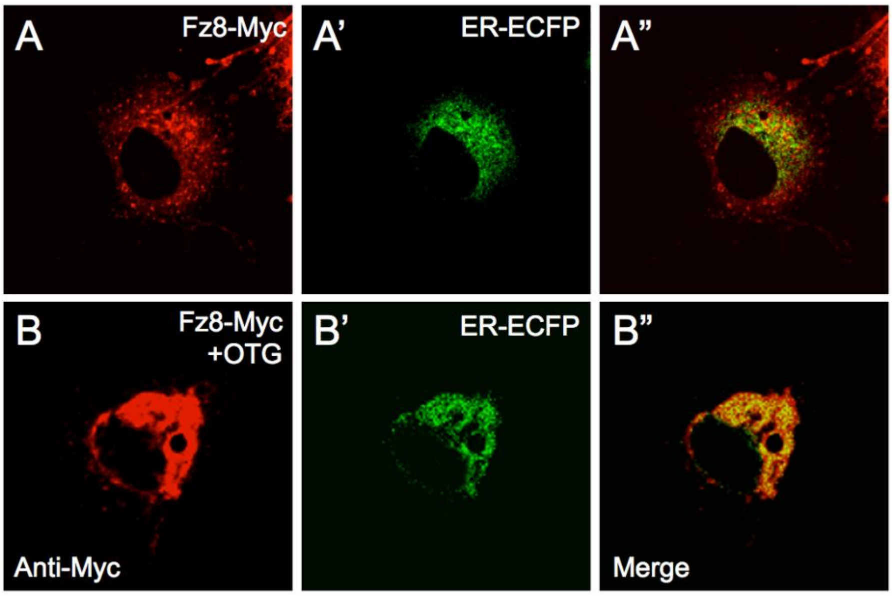

Supplementary information, Figure S12. OTG restricts the distribution Fz8 to the ER in mammalian cells

(A-B"). Immunostaining of COS-7 cells transfected for Fz8-Myc and ER-ECFP alone (A-A") or with OTG (B-B"). Overexpressed Fz8-Myc (red) is visualized by anti-Myc immunostaining. ER-ECFP (green) is used as an exogenous ER marker. Only a portion of Fz8-Myc is localized in the ER in OTG un-transfected cells (A-A") whereas majority of Fz8-Myc is localized in the ER in OTG transfected cells (B-B").

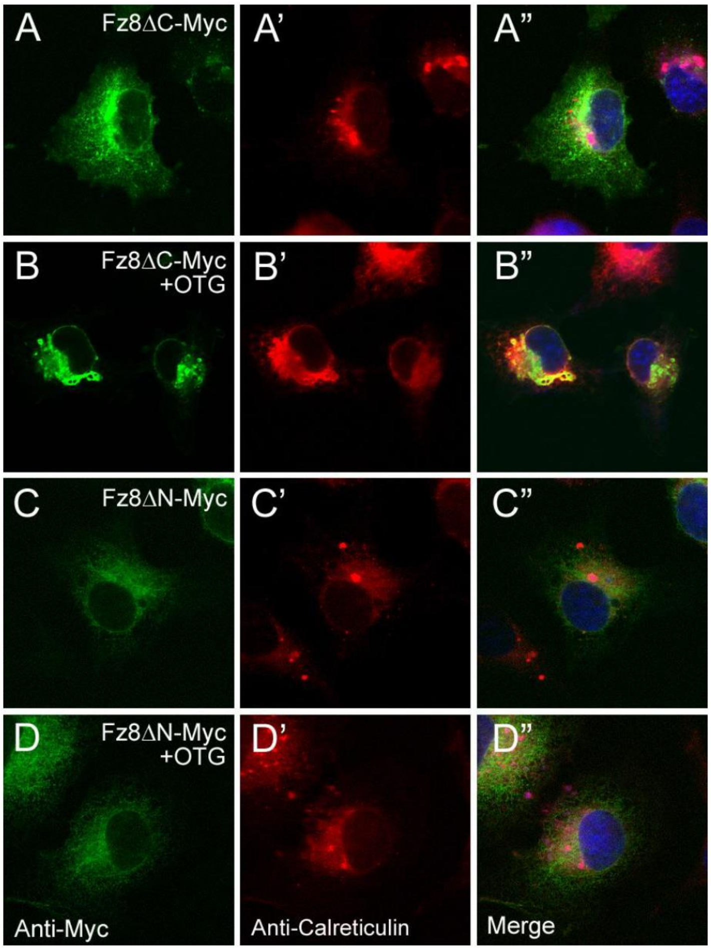

Supplementary information, Figure S13. The N-terminus of Fz8 is required for OTG-induced ER retention

(A-D") Immunostaining of COS-7 cells transfected for indicated Fz8-Myc deletion construct alone (A-A", C-C") or with OTG (B-B", D-D"). Overexpressed Fz8-myc proteins (green) are visualized by anti-Myc immunostaining along with calreticulin (red). Nuclei are counter-stained with Hoechst 33342 (blue). OTG induces ER retention of Fz8ΔC-Myc (B-B") whereas OTG fails to do so for Fz8ΔN-Myc (D-D").

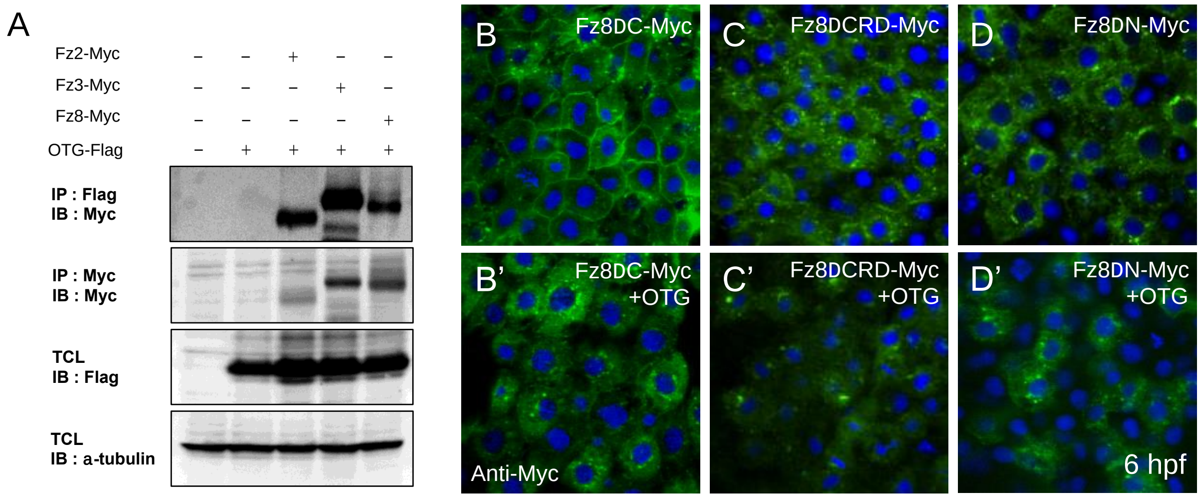

Supplementary information, Figure S14. The N-terminus of Fz8 is required for OTG-induced change of Fz8 subcellular localization

(A) Co-immunoprecipitation of OTG-Flag with Fz2-Myc, Fz3-Myc, and Fz8-Myc after co-transfection. (B-D') Immunostaining of gastrula embryos injected with mRNA for indicated *fz8-myc* deletion mutant alone (B, C, D) or with *OTG* mRNA (B', C,' D'). Overexpressed Fz8-myc proteins (green) are visualized by anti-Myc immunostaining. Nuclei are counter-stained with Hoechst 33342 (blue). Subcellular distribution of Fz8ΔC-Myc and Fz8ΔCRD-Myc are affected upon co-expression of OTG. However, OTG fails to change the subcellular distribution of Fz8ΔN-Myc.

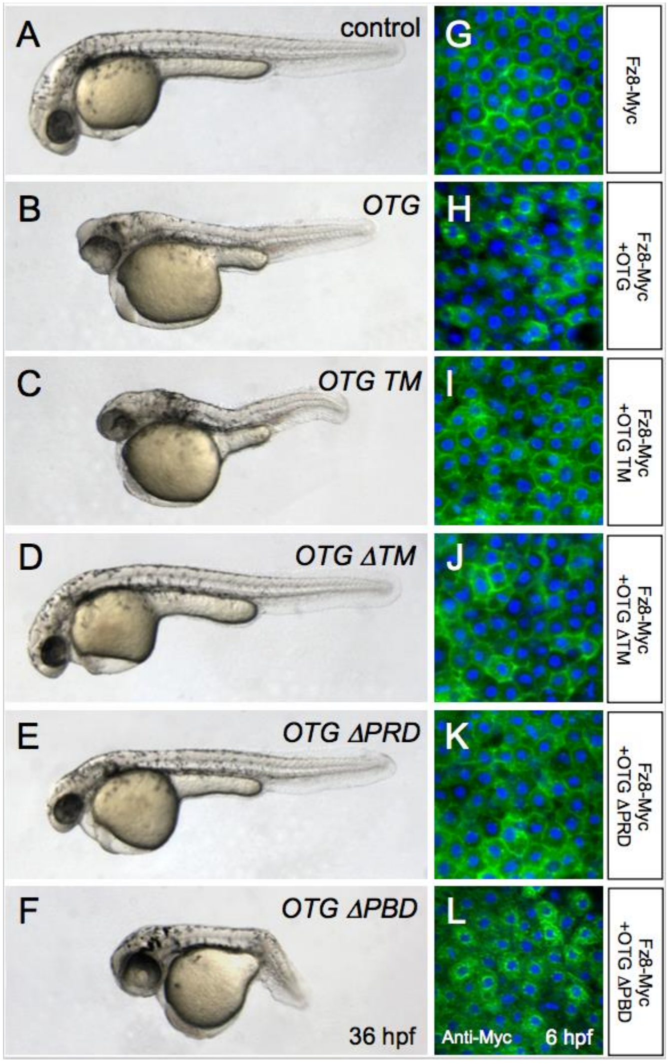

Supplementary information, Figure S15. Effects of various *OTG* deletion mutants in zebrafish development

(A-F) Morphology (at 36 hpf) of control embryo (A, 12/12, 100%) and embryos injected with mRNA for indicated *OTG* deletion mutant. Overexpression of OTGΔPBD-Flag results enlarged head and shortened tail (F, 15/16, 94%) similar to wild type OTG (B, 15/15, 100%). OTG TM-Flag (C, 10/14, 71%) and OTGΔPRD-Flag (E, 12/16, 75%) lead to small head with normal tail. OTGΔTM-Flag does not cause any discernible defect.

(G-L) Immunostaining of overexpressed Fz8-Myc (green) in shield stage embryos injected with mRNA for *fz8-myc* alone (G) or with mRNA for indicated *OTG-Flag* deletion mutant. Overexpressed Fz8-myc proteins (green) are visualized by anti-Myc immunostaining. Nuclei are counter-stained with Hoechst 33342 (blue). Cell surface expression of Fz8-Myc is affected by OTG-Flag (H) and OTGΔPBD-Flag (L). However, OTG TM-Flag (I), OTGΔTM-Flag (J), and OTGΔPRD-Flag (K) do not affect the membrane localization of Fz8-Myc.
